# Unveiling Metabolic Engineering Strategies by Quantitative Heterologous Pathway Design

**DOI:** 10.1002/advs.202404632

**Published:** 2024-10-16

**Authors:** Fan Wei, Jingyi Cai, Yufeng Mao, Ruoyu Wang, Haoran Li, Zhitao Mao, Xiaoping Liao, Aonan Li, Xiaogui Deng, Feiran Li, Qianqian Yuan, Hongwu Ma

**Affiliations:** ^1^ Biodesign Center Key Laboratory of Engineering Biology for Low‐carbon Manufacturing Tianjin Institute of Industrial Biotechnology Chinese Academy of Sciences Tianjin 300308 China; ^2^ National Technology Innovation Center for Synthetic Biology Tianjin 300308 China; ^3^ University of Chinese Academy of Sciences Beijing 100049 China; ^4^ School of Biological Engineering Tianjin University of Science and Technology Tianjin 300457 China; ^5^ Institute of Biopharmaceutical and Health Engineering Tsinghua Shenzhen International Graduate School Tsinghua University Shenzhen 518055 China

**Keywords:** engineering strategies, metabolic network, pathway design

## Abstract

Constructing efficient cell factories requires the rational design of metabolic pathways, yet quantitatively predicting the potential pathway for breaking stoichiometric yield limit in hosts remains challenging. This leaves it uncertain whether the pathway yield of various products can be enhanced to surpass the stoichiometric yield limit and whether common strategies exist. Here, a high‐quality cross‐species metabolic network model (CSMN) and a quantitative heterologous pathway design algorithm (QHEPath) are developed to address this challenge. Through systematic calculations using CSMN and QHEPath, 12,000 biosynthetic scenarios are evaluated across 300 products and 4 substrates in 5 industrial organisms, revealing that over 70% of product pathway yields can be improved by introducing appropriate heterologous reactions. Thirteen engineering strategies, categorized as carbon‐conserving and energy‐conserving, are identified, with 5 strategies effective for over 100 products. A user‐friendly web server is developed to quantitatively calculate and visualize the product yields and pathways, which successfully predicts biologically plausible strategies validated in literature for multiple products.

## Graphical abstract

The Quantitative Heterologous Pathway Design method (QHEPath) is developed to evaluate 12,000 biosynthetic scenarios across 300 products and 4 substrates in 5 industrial organisms. This method reveals that over 70% of product yields can be improved with heterologous reactions. Thirteen engineering strategies are identified, and a web server is created to quantitatively calculate and visualize yields and pathways.

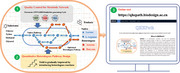

## 1 Introduction

Microbial cell factories with efficient biosynthetic pathways enable the production of various chemicals from renewable carbon sources.^[^ ^1^ ^]^ Over the past decades, these cell factories have successfully produced a diverse range of chemical compounds,^[^ ^2^ ^]^ including biofuels,^[^ ^3^ ^]^ platform chemicals,^[^ ^4^ ^]^ pharmaceuticals,^[^ ^5^ ^]^ and food additives.^[^ ^6^ ^]^ Pathway yield (*Y^P^*) is the amount of a product formed from a substrate, computed based on the stoichiometry of the host.^[^ ^7^ ^]^ *Y^P^* is a crucial metric for designing efficient and atom‐economical cell factories. Previous reports have indicated that introducing heterologous pathways was effective in enhancing *Y^P^* to break the yield limit in a host.^[^ ^8^ ^]^ For example, the *Y^P^* of farnesene in the final engineered strain broke the yield limit of the native network stoichiometry by adding the heterologous non‐oxidative glycolysis (NOG) pathway.^[^ ^9^ ^]^ Additionally, our laboratory has reported that introducing the NOG pathway was effective in enhancing poly(3‐hydroxybutyrate) (PHB) yield to exceed the yield limit in *E. coli*.^[^ ^8b^ ^]^ However, these studies focus on specific products and primarily rely on experience to determine heterologous reactions without developing computational methods and summarizing common strategies. Hence, there is a need to develop a rational computational method and conduct extensive studies to determine whether the *Y^P^* of a wide range of products can be enhanced to break the yield limits of hosts and whether there are common strategies effective across various products and hosts.

Genome‐scale metabolic models (GEMs) comprehensively represent an organism's metabolism, integrating all metabolic reactions annotated from its genome.^[^ ^10^ ^]^ *Y^P^* can be calculated using the GEM of a strain through flux balance analysis (FBA).^[^ ^11^ ^]^ Individual species GEMs only incorporate species‐specific reactions, limiting the calculation of pathways for products that cannot be naturally synthesized. For example, lycopene cannot be naturally synthesized by *E. coli*, so its synthesis pathway and yield cannot be directly calculated using the GEM of *E. coli*. Additionally, the diversity of reactions in individual species GEMs is limited, restricting the exploration of heterologous pathway introductions to enhance *Y^P^*. Constructing an extensive metabolic space that encompasses a diverse array of biochemical reactions across multiple species becomes imperative. Previous studies have attempted to expand the GEMs of individual species by incorporating reactions from cross‐species biochemical reaction databases to construct integrated models. For example, some studies^[^ ^12^ ^]^ integrated the GEMs of *E. coli* and *S. cerevisiae* with the KEGG database^[^ ^13^ ^]^ to construct the integrated models. The models were utilized to calculate the minimum number of heterologous reactions required for non‐native product synthesis. Similarly, Chatsurachai et al.^[^ ^14^ ^]^ expanded the GEMs of three organisms by sequentially adding heterologous reactions from metabolic databases. Under the guidance of the integrated model, it is possible to identify producible pathways for any non‐native product in three hosts. These studies utilized integrated models solely to identify heterologous reactions for the producibility of non‐native products, without considering the introduction of heterologous reactions to further improve *Y^P^*. When we evaluated these published integrated models, we found an error that is the pathway maximum yield ($Y_{m}^{P}$), determined as the maximum amount of a product formed from a substrate while considering the large‐scale stoichiometries network of integrated models, exceeded the theoretical maximum yield (*Y^E^*) calculated by the reduction degrees of the substrate and the product.^[^ ^12a^ ^]^ This error was caused by the infinite generation of reducing equivalents in the integrated models. Therefore, to obtain the correct $Y_{m}^{P}$, it is necessary to perform quality control on the integrated models to eliminate these errors. While several methods have been developed for testing (MEMOTE^[^ ^15^ ^]^) and fixing (G_LOBA_LF_IT_^[^ ^16^ ^]^) errors in GEMs, they are powerless in automatically identifying and rectifying a multitude of errors with infinite generation of reducing equivalents, energy, and metabolites (Note S1, Supporting Information). Hence, it is urgent to develop a standardized automated quality‐control workflow for eliminating these errors in the integrated model.

In addition to high‐quality integrated models, algorithms are also essential for assessing enhancements in *Y^P^* and identifying the necessary heterologous reactions required to exceed the yield limit in a host. We use the producibility yield (*Y* ^*P*0^) as the yield limit of a product from a substrate in a host without introducing any heterologous reactions except for the minimal set of heterologous reactions essential for the producibility of non‐native products. The evaluation of whether *Y* ^*P*0^ can be surpassed by introducing heterologous reactions in a host, can be determined by comparing the increase in $Y_{m}^{P}$ relative to *Y* ^*P*0^. Existing heterologous pathway design algorithms, such as OptStrain,^[^ ^17^ ^]^ were designed to compute the minimum heterologous reactions required to be introduced into the host model to reach $Y_{m}^{P}$. However, for non‐native products, OptStrain cannot distinguish between the reactions responsible for reaching *Y* ^*P*0^ and those contributing to reaching $Y_{m}^{P}$. This makes it difficult to identify specific heterologous reactions that contribute to breaking the yield limit of the host. Furthermore, for breaking the yield limit *Y* ^*P*0^, there may be multiple potential strategies for introducing heterologous reactions. However, OptStrain does not consider the possibilities involving fewer heterologous reactions at suboptimal yields.^[^ ^18^ ^]^ Consequently, developing a new quantitative heterologous pathway design algorithm is essential.

In this study, a quality‐control workflow was created to refine all reactions in the BiGG database,^[^ ^19^ ^]^ enabling the construction of a high‐quality cross‐species metabolic model (CSMN) by automatically eliminating various types of errors. Then, a quantitative heterologous pathway design algorithm (QHEPath) was devised to explore the heterologous reactions for enhancing *Y^P^* to break the yield limit *Y* ^*P*0^. Employing the model CSMN and the algorithm QHEPath, the enhancement of *Y^P^* for 300 value‐added chemicals across 15 diverse categories in multiple industrial microbial species was investigated. Through the analysis of multiple heterologous pathways for various products, various engineering strategies for breaking the yield limit were summarized, and some of them were verified by the literature. In‐depth investigations of the synthesis precursors and surplus of reducing equivalents were conducted to understand the characteristics and selection of these strategies. To enhance the accessibility for biologists, the first user‐friendly web server for quantitative heterologous pathway design, QHEPath (https://qhepath.biodesign.ac.cn/), has been developed. It effectively predicts biologically feasible strategies that have been validated in the literature for various products.

## 2 Results

### 2.1 Reconstruction of a Cross‐Species Metabolic Network Model

**Figure 1:**
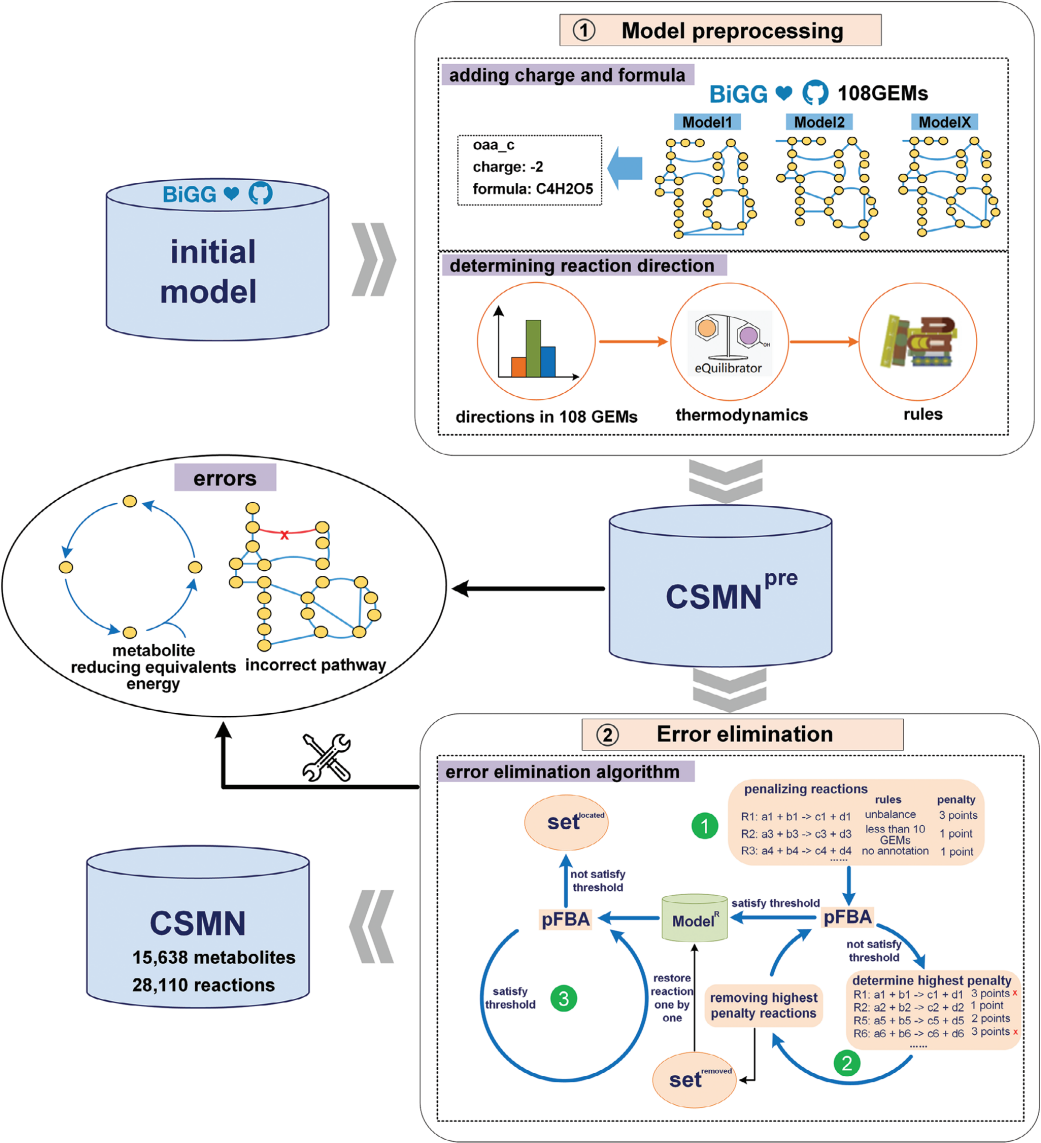
The workflow of reconstructing the cross‐species metabolic network (CSMN) model. The workflow uses the universal model from the BiGG database as the initial model. This initial model is preprocessed, which involved integrating metabolite charge and formula information and determining reaction directions (Step 1). After preprocessing, the CSMN^pre^ model was generated; however, it contained various errors. The error elimination algorithm was then applied for error correction (Step 2). Upon the removal of these errors, the high‐quality CSMN model was reconstructed.

The cross‐species metabolic network (CSMN) model was reconstructed based on the universal model from the BiGG database.^[^ ^19^ ^]^ The universal model contained 15638 metabolites and 28301 reactions spanning 108 GEMs across 35 species, providing broad coverage of common industrial species. It has the potential to serve as an integrated model for predicting the improvements of *Y^P^* with flexible switch of hosts. However, when the initial universal model was used to calculate $Y_{m}^{P}$ with glucose as the substrate, most $Y_{m}^{P}$ values for carbon‐containing metabolites exceeded *Y^E^* (Table S4, Supporting Information Data 1). For example, the yield of acetate was calculated to be 100 mol mol^−1^ glucose by the initial universal model, exceeding *Y^E^* of 3 mol mol^−1^ glucose. This indicated errors within the model that require quality control. Here, we developed a quality‐control workflow to refine reactions in the initial universal model to reconstruct the high‐quality CSMN. The reconstruction process involved preprocessing and eliminating errors in the initial universal model (**Figure** **1**). The initial universal model lacked crucial details such as metabolite charge, formula information, and reaction directions. The preprocessing included incorporating metabolite charge and formula information into the model and determining the directions of the reactions (Figure 1, step 1). The charge and formula of the metabolites were extracted from the 108 GEMs of the BiGG database. The direction of the reaction was initially determined by counting their occurrences in the 108 GEMs. For reactions appearing in a limited number of GEMs, corrections were made for potential inaccuracies. The direction of these reactions was corrected based on thermodynamics and heuristic rules. Details of reaction direction correction were provided in the Methods. Specifically, 287 reaction directions were corrected using Gibbs free energy, and 271 reaction directions were corrected based on heuristic rules^[^ ^20^ ^]^ (Tables S1 and S2, Supporting Information Data 1). After the preprocessing, the CSMN^pre^ model was obtained.

After model preprocessing, it was observed that $Y_{m}^{P}$ of all carbon‐containing metabolites exceeded *Y^E^* with glucose as the substrate in the CSMN^pre^ model (Table S4, Supporting Information Data 1). This indicated that the CSMN^pre^ model was unable to accurately calculate $Y_{m}^{P}$. Further analysis revealed errors in the model, including the infinite generation of metabolites, reducing equivalents, energy without substrate supply, and erroneous pathways (Figure S1, Supporting Information). Given the complexity of the metabolic network, manual identification and rectification of these errors is challenging.^[^ ^21^ ^]^ Therefore, a novel automated error elimination method based on parsimonious enzyme usage FBA (pFBA)^[^ ^22^ ^]^ was developed (Figure 1, step 2). The method encompassed the following three main steps: 1) penalizing reactions, 2) iteratively applying pFBA with an objective function and threshold to remove high‐penalty reactions until the threshold is satisfied, and 3) sequentially restoring the removed reactions to pinpoint the specific reaction responsible for the error (Figure 1, step 2) (details in Experimental Section).

By employing the automated error elimination method for the CSMN^pre^ model, various types of errors were effectively rectified by setting distinct objective functions and thresholds (Figure 1, step 2). Among the reactions associated with various errors, 45 reactions with mass unbalances were curated, 12 reactions with incorrect directions were revised, and 175 reactions displaying mass unbalances, including those with macromolecular, biomass, or no annotation information, were removed (Table S3, Supporting Information Data 1). Following the elimination of all errors, the final model CSMN was reconstructed, encompassing 15638 metabolites and 28110 reactions. CSMN can be effectively utilized for correct product yield calculations, ensuring that $Y_{m}^{P}$ of all metabolites calculated by the model CSMN does not surpass *Y^E^* (Table S4, Supporting Information Data 1).

### 2.2 Development of the Quantitative Heterologous Pathway Design Algorithm

To investigate whether *Y^P^* can be improved by introducing heterologous reactions, it is necessary to calculate $Y_{m}^{P}$ in the CSMN and *Y* ^*P*0^ in the host model. If the $Y_{m}^{P}$ in the CSMN is greater than the *Y* ^*P*0^ in the host model, it indicates that the yield limit *Y* ^*P*0^ can be broken by introducing heterologous reactions. For native products, *Y* ^*P*0^ can be directly determined using FBA. However, for non‐native products, it is essential to first determine the minimal introduction of heterologous reactions required for producibility. To achieve this goal, a quantitative heterologous pathway design algorithm (QHEPath) was developed to directly calculate the improvement of *Y^P^* for native and non‐native products by introducing the minimal number of heterologous reactions into the chassis. However, some products require the introduction of multiple heterologous reactions to achieve *Y^P^* reaching $Y_{m}^{P}$. For example, spermidine, sarcosine, and 4‐hydroxy‐benzyl alcohol require the introduction of 7, 6, and 12 heterologous reactions, respectively, to reach $Y_{m}^{P}$ (Table S6, Supporting Information Data 1). In the genetic modification of metabolic engineering, introducing additional heterologous reactions may result in a metabolic burden on the cells. Pathways that involve the introduction of fewer heterologous reactions to achieve suboptimal yields might be more attractive. Therefore, it is necessary to trade‐off between the improvement of *Y^P^* and the number of heterologous reactions in the QHEPath algorithm.

**Figure 2:**
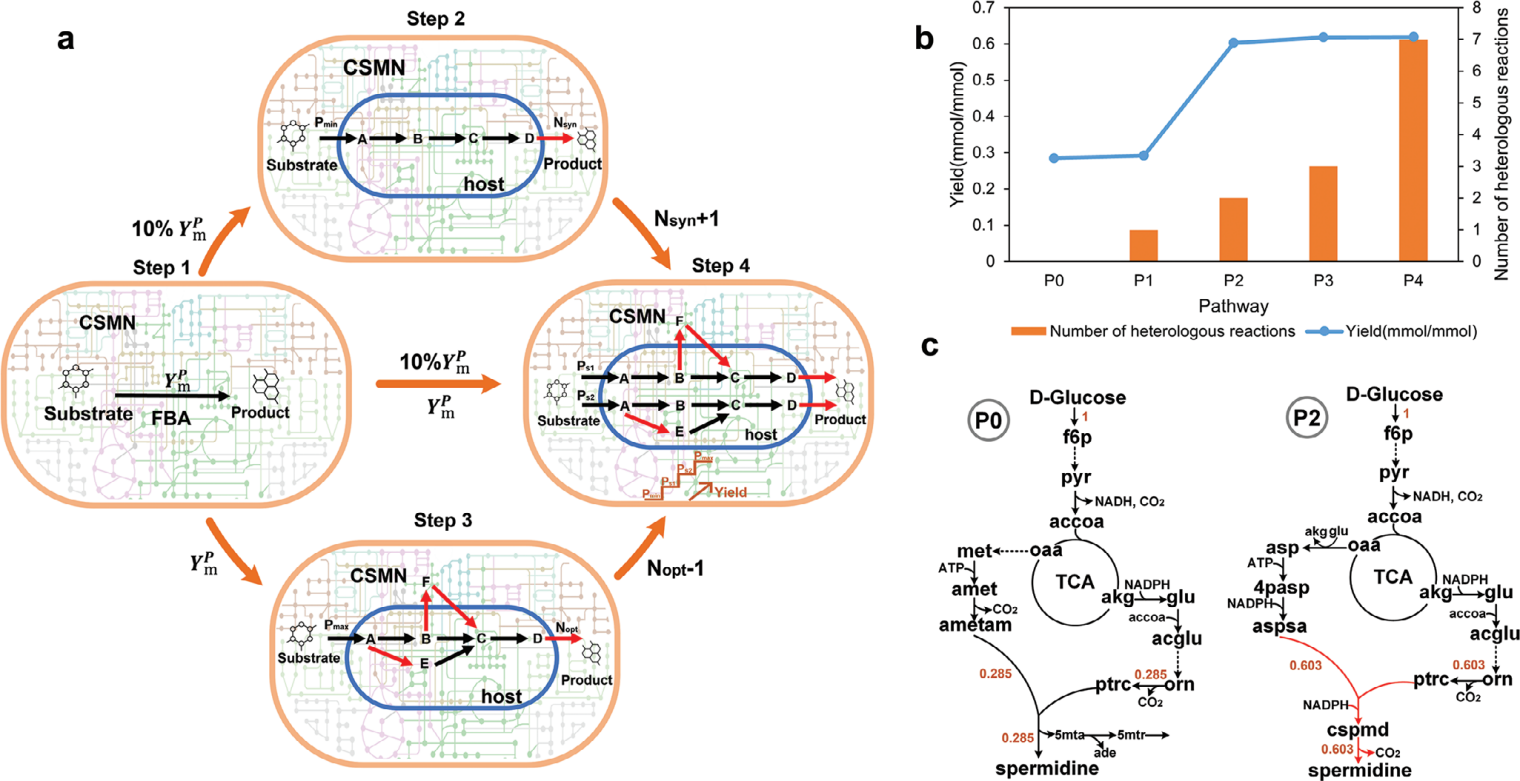
Quantitative heterologous pathway design algorithm (QHEPath) and the synthesis pathway of spermidine. a) Schematic for the algorithm QHEPath. b) The yield of the native spermidine synthesis pathway P0 and multiple pathways P1–P4 with higher yields than P0 were obtained by the introduction of heterologous reactions using *E. coli* as the chassis. c) The native biosynthetic pathway P0 of spermidine and pathway P2 with a higher yield achieved by the introduction of two heterologous reactions. Black arrows represent native reactions and red arrows represent heterologous reactions. The numbers on the pathway diagram represent reaction flux. pyr, pyruvate; accoa, acetyl‐CoA; oaa, oxaloacetate; akg, 2‐oxoglutarate; glu, L‐glutamate; acglu, N‐acetyl‐L‐glutamate; orn, ornithine; ptrc, putrescine; met, L‐methionine; amet, S‐adenosyl‐L‐methionine; ametam, S‐adenosylmethioninamine; 5mta, 5‐methylthioadenosine; 5mtr, 5‐methylthio‐D‐ribose; ade, adenine; asp, L‐Aspartate; 4pasp, 4‐phospho‐L‐aspartate; aspsa, L‐aspartate 4‐semialdehyde; cspmd, carboxyspermidine.

The algorithm QHEPath comprised four steps (**Figure** **2**a): 1) calculating the maximum pathway yield ($Y_{m}^{P}$) in the model CSMN by FBA; 2) determining the minimum number of heterologous reactions (N_syn_) to enable product producibility when the *Y^P^* is at least 10% of $Y_{m}^{P}$ for non‐native products. Native products can achieve product producibility without the introduction of heterologous reactions, and N_syn_ is 0. 3) calculating the minimum number of heterologous reactions (N_opt_) to achieve optimal product synthesis when the *Y^P^* reaches the $Y_{m}^{P}$; 4) carrying out the stepwise introduction of heterologous reactions from N_syn_ +1 to N_opt_ −1 to obtain multiple suboptimal pathways (details in Methods). Moreover, to assess the parameter stability and computational efficiency of the QHEPath algorithm, we conducted additional tests, with detailed results provided in Note S4 (Supporting Information).

The QHEPath algorithm not only determines *Y* ^*P*0^ and $Y_{m}^{P}$ but also computes multiple suboptimal and optimal pathways. The model CSMN and the algorithm QHEPath were utilized to calculate suboptimal and optimal pathways for spermidine in *E. coli* using glucose as the substrate. Spermidine is a crucial polyamine with applications in the synthesis of diverse bioactive compounds in the food and pharmaceutical fields.^[^ ^23^ ^]^ In the case of spermidine synthesis, the endogenous pathway in *E. coli* exhibits a low yield due to the inability to reuse intermediates 5‐methylthio‐D‐ribose (5mtr) (Figure 2c, P0). QHEPath identified four pathways (P1∼P4) with higher yields than the native pathway P0 to surpass the yield limit (Figure 2b). Notably, with the continuous introduction of heterologous pathways, the *Y^P^* consistently improved. Interestingly, upon the introduction of two heterologous reactions (P2), the yield of spermidine reaches an impressive 97% (0.603 mmol mmol^−1^) of $Y_{m}^{P}$ (0.619 mmol mmol^−1^), representing a remarkable 118% increase compared to the native pathway P0 (Figure 2b). Pathway P2 introduced two heterologous reactions to circumvent the generation of 5mtr, redirecting more carbon flux toward the target product spermidine (Figure 2c). This pathway has also been experimentally validated for the efficient synthesis of spermidine in *E. coli*.^[^ ^24^ ^]^ Pathway P4 exhibited a higher yield than P2 and reached $Y_{m}^{P}$, but it required the introduction of seven heterologous reactions, resulting in only a 3% increase in yield. Additionally, the synthetic pathways for some products, such as sarcosine and 4‐hydroxy‐benzyl alcohol, predicted by the model and the QHEPath algorithm, also require balancing yield improvement and the number of heterologous reactions (details in Note S5, Supporting Information). Therefore, this algorithm QHEPath plays a crucial role in selecting a more biologically realistic and efficient pathway to break the yield limit in a host for metabolic engineering modifications, requiring fewer genetic manipulations.

### 2.3 Enhancing Product Yields Through Quantitative Heterologous Pathway Design

**Figure 3:**
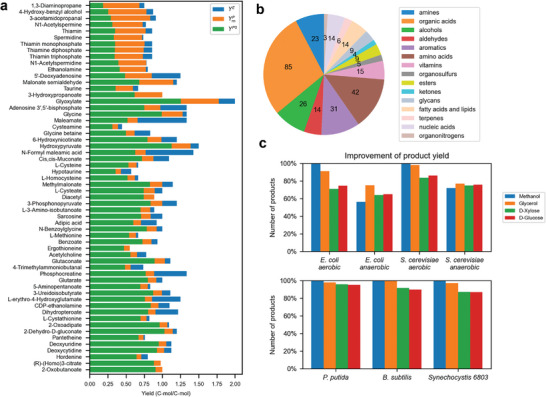
The improvement in pathway yields for 300 products across different species, and substrates under aerobic and anaerobic conditions. a) Products exhibiting at least a 10% increase when glucose is the carbon source in *E. coli*. The green bar represents the yield of product producibility (*Y* ^*P*0^) in *E. coli*. The orange bar represents that *Y^P^* reaches $Y_{m}^{P}$ by introducing a minimal number of heterologous reactions. The blue bar represents the maximum theoretical yield *Y^E^*. b) Classification of 300 products according to their chemical characteristics. The numbers on the pie chart indicate the number of products in each category. c) The percentage of the number of products with improved yields across different substrates and species under aerobic and anaerobic conditions. The facultative anaerobes were considered for *E. coli* and *S. cerevisiae* and the obligate aerobes were assumed for *P. putida*, *B. subtilis*, and *Synechocystis* 6803.

To test the performance of the CSMN model and the QHEPath algorithm in predicting product yield improvements. A total of 300 value‐added products were collected from the literature.^[^ ^12^, ^25^ ^]^ These products spanned 15 diverse categories, including amino acids, aromatic compounds, terpenes, and various other classes (**Figure** **3**b). Given its widespread use as a model organism, *E. coli* was first chosen as the chassis for the calculation of these products.^[^ ^26^ ^]^ Among the 300 products, 123 were identified as native, and 177 were non‐native based on the calculation with the metabolic model of *E. coli*, iML1515.^[^ ^27^ ^]^ As the QHEPath algorithm can directly determine the improvement of *Y^P^*, it was observed that the incorporation of heterologous reactions led to enhanced *Y^P^* for 75% (224/300) of the products when glucose was used as the carbon source (Table S5, Supporting Information Data 1). Most products required the introduction of up to seven heterologous reactions to enhance *Y^P^* (Figure S2, Supporting Information). Among these products, 59 exhibited yield improvements exceeding 10% (Figure 3a). However, the yields of 76 products have not been improved (Table S5, Supporting Information Data 1). Among these, 37 products have already reached *Y^E^*, leaving no room for further improvement. The remaining 39 products have not shown yield improvement, likely due to the lack of suitable heterologous reactions in the CSMN model to enhance product yields.

To provide a more comprehensive understanding of the potential for enhancing *Y^P^* to exceed *Y* ^*P*0^ by introducing heterologous pathways, the study explored different substrates and species. Four substrates (methanol, glycerol, D‐xylose, D‐glucose) and five representative organisms, widely employed as cell factories in numerous biotechnological applications spanning prokaryotes and eukaryotes, were chosen.^[^ ^28^ ^]^ The organisms, along with their associated established GEMs in the BiGG database, were as follows: *E. coli* (iML1515^[^ ^27^ ^]^), *Pseudomonas putida* (iJN1463^[^ ^29^ ^]^), *Saccharomyces cerevisiae* (iMM904^[^ ^30^ ^]^), *Bacillus subtilis* (iYO844^[^ ^31^ ^]^), and *Synechocystis* sp. PCC 6803 (iSynCJ816^[^ ^32^ ^]^). The improvements of *Y^P^* were calculated in 12000 biosynthetic scenarios, including 300 products from four substrates in five industrial organisms under aerobic/anaerobic conditions using the model CSMN and the algorithm QHEPath (Supporting Data 2). It was worth noting that for over 70% of the products, yield improvements were achievable when different substrates and species were considered under aerobic conditions (Figure 3c). Specifically, when methanol was used as the substrate, the yields of all products were increased. Furthermore, product yields were highest in different hosts when methanol was used as the substrate (Figure S3, Supporting Information). This was mainly due to methanol having the highest carbon molar reduction degree (calculated based on the ratio of the reduction degree of the metabolite to the number of carbon atoms) among these four substrates. Consequently, more surplus electrons are available, contributing to the improvement of *Y^P^* by introducing heterologous reactions from the model CSMN. Methanol could potentially serve as an attractive substrate for biotechnological applications. Furthermore, the analysis was extended to examine the potential for enhancing *Y^P^* through the introduction of heterologous pathways in both *E. coli and S. cerevisiae* under anaerobic conditions. Under anaerobic conditions, only 240 of the 300 products can be synthesized in *E. coli* and *S. cerevisiae* due to oxygen requirements, and the percentages were calculated based on the 240 products. We found the yields of at least 70% of the products were enhanced by introducing appropriate heterologous reactions in *E. coli*, over 90% of the products showed improved yields in *S. cerevisiae* (Table S1, Supporting Information). Hence, these results demonstrate that yield limit *Y* ^*P*0^ of most products can be broken in different hosts, substrates, and oxygen supplying conditions.

To assess the differences for synthesizing diverse products in different hosts, we calculated the minimum number of heterologous reactions and synthetic yields for 15 categories of products in these 5 hosts. Given that different hosts inherently possess diverse metabolic pathways, the heterologous reactions required for product synthesis also vary. For example, terpenes can be naturally synthesized in *Synechocystis* sp. PCC 6803 (iSynCJ816) without the introduction of any heterologous reactions (Supporting Information, Figure S4a, Supporting Information). However, in other hosts, multiple heterologous reactions are needed for terpenes synthesis. Additionally, different hosts exhibit varying yields for the same type of products, providing a reference for selecting the appropriate chassis for product synthesis. For instance, organosulfur, nucleic acids, and organonitrogens have the lowest yields in the host *Pseudomonas putida* (iJN1463) compared to other hosts (Supporting Information, Figure S4b, Supporting Information), indicating that *Pseudomonas putida* may not be the ideal chassis for synthesizing these three types of products. In summary, these results highlight the differences in metabolic pathways among various hosts, providing researchers with a systematic and data‐driven approach to select the optimal host for producing specific products with higher yields and lower costs.

### 2.4 Strategies for Enhancing the Pathway Yield

By analyzing the suboptimal and optimal pathways for 300 products, it was observed that certain heterologous reactions recurred in the optimization pathways of multiple products (Tables S6 and S7, Supporting Information Data 1). To further assess the significance of these heterologous reactions, a statistical analysis of their frequency in the obtained suboptimal and optimal pathways was conducted (see Table S8, Supporting Information Data 1). 76 heterologous reactions were identified in at least five product optimization pathways. These reactions were integrated individually or in combination into the *E. coli* model iML1515 to evaluate their effectiveness in enhancing *Y^P^* of multiple products. Employing heterologous reactions to enhance the yield of at least five products was regarded as a viable strategy. A total of 13 strategies (S1–S13) were screened out and summarized in **Table** **1**.

**Table 1:** Thirteen strategies for enhancing the pathway yield from Figure 5 and the average improvement of pathway yields under different oxygen conditions.

Strategy	Heterologous reactions	Improvement of pathway yields [%]		
		Aerobic	Microaerobic	Anaerobic
S1	PRUK: atp + ru5p__D = > adp + h + rb15bp, RBPC: co2 + h2o + rb15bp = > 2.0 3pg + 2.0 h	14.38	10.97	12.12
S2	THRA: thr__L = > acald + gly, THRD: nad + thr__L = > 2aobut + h + nadh	14.62	10.76	12.07
S3	OOR2r: akg + coa + fdxo_42 <=> co2 + fdxr_42 + h + succoa, FRDO6r: fdxr_42 + h + nad <=> fdxo_42 + nadh	18.90	16.94	23.17
S4	2DHPFALDL:2dhp <=> 3mob + fald, AH6PI: ah6p__D = > f6p, RU5PS: fald + ru5p__D = > ah6p__D	13.49	14.16	19.60
S5	PKETF: f6p + pi = > actp + e4p + h2o, PKETX: pi + xu5p__D = > actp + g3p + h2o	16.45	12.61	17.10
S6	SSCOARy:h + nadph + succoa = > coa + nadp + sucsal, OCOAT3r: ghb + succoa <=> 4hbutcoa + succ, VACOAI: vaccoa = > b2coa, 4HBCOAH: 4hbutcoa = > h2o + vaccoa	20.39	12.57	15.90
S7	AB3CL: ab3coa + h = > b2coa + nh4, DH36M: 36dahx = > dah35 + h, DH35O: dah35 + h2o + nad = > a53oh + nadh + nh4, A53C: a53oh + accoa + 3.0 h = > ab3coa + acac	15.98	13.41	16.56
S8	PFK_ppi: f6p + ppi <=> fdp + h + pi	2.69	11.29	12.74
S9	ANS2: chor + nh4 = > anth + h2o + h + pyr	1.92	9.56	10.21
S10	ORNTAC: acorn + glu__L <=> acglu + orn	1.96	8.67	17.83
S11	HMGCOAS:coa + h + hmgcoa <=> aacoa + accoa + h2o, HMGCOAR: coa + mev__R + 2.0 nadp <=> 2.0 h + hmgcoa + 2.0 nadph, PMEVK:5pmev + atp = > 5dpmev + adp, DPMVD:5dpmev + atp = > adp + co2 + ipdp + pi, MEVK1x: atp + mev__R = > 5pmev + adp + h	2.88	10.56	7.06
S12	HSDA: hom__L = > 2obut + nh4	3.38	11.13	16.06
S13	SADT: atp + h + so4 = > aps + ppi, APSR2: aps + grxrd = > amp + grxox + 2.0 h + so3	4.21	9.92	16.24

**Figure 4:**
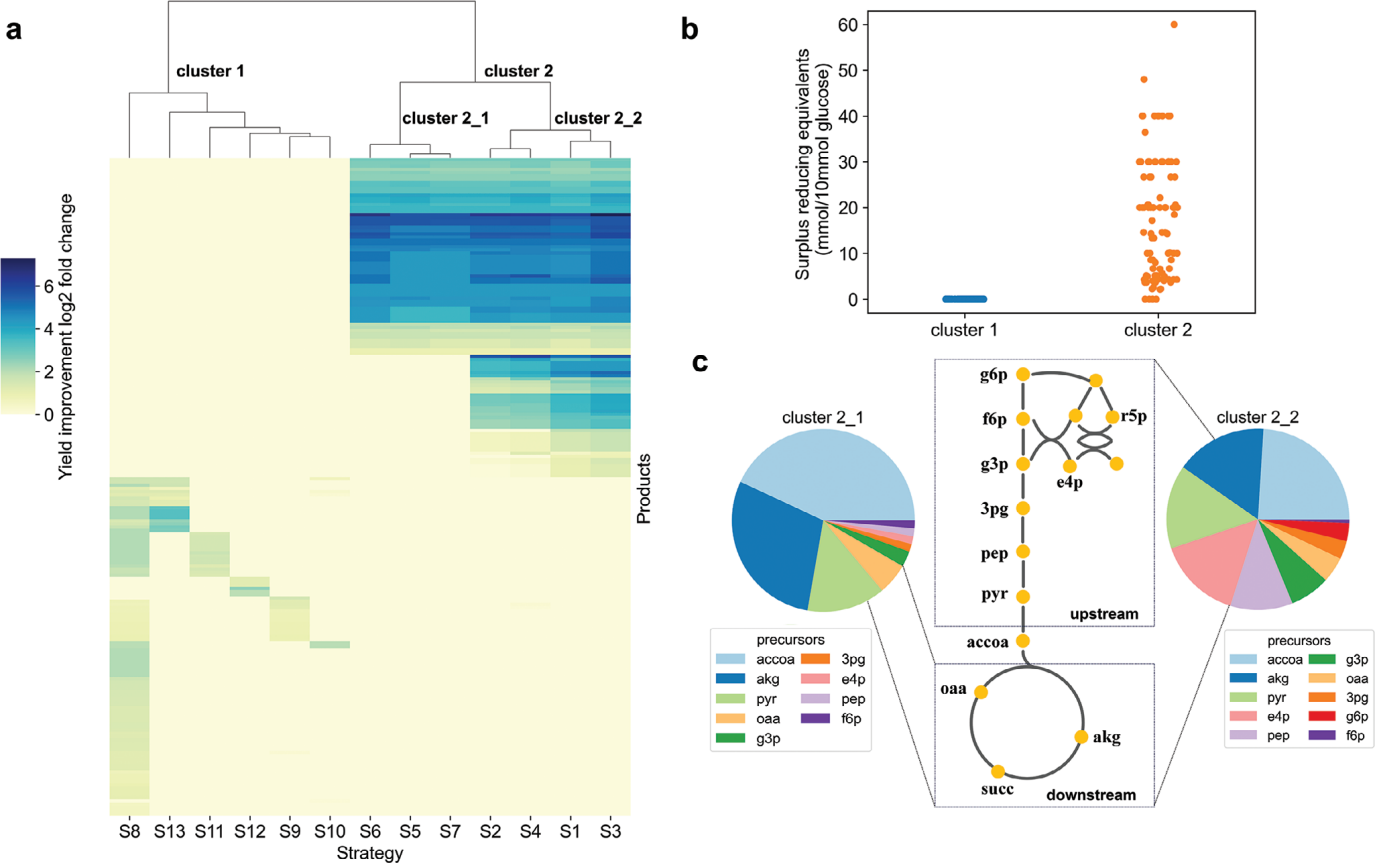
Clustering and characteristic analysis of 13 strategies. a) Hierarchical cluster analysis for 13 strategies. b) The surplus of reducing equivalents varies among different clusters. c) There are 12 precursors in central carbon metabolism involved in product synthesis, and the specific precursors are primarily utilized in each cluster.

To systematically analyze strategy characteristics and guide their selection, hierarchical clustering of these strategies was performed. Notably, these strategies were divided into two main clusters (cluster 1 and cluster 2) (**Figure** **4**a) (Table S9, Supporting Information Data 1). Cluster 1 comprised strategies S8 to S13, while Cluster 2 encompassed strategies S1 to S7. To explore the characteristics of products associated with these strategies, we analyzed the surplus of reducing equivalents and synthetic precursors in these product pathways (Table S9, Supporting Information Data 1). It can be observed that the products in cluster 2 exhibited a surplus of reducing equivalents, whereas the products in cluster 1 did not show any surplus of reducing equivalents (Figure 4b). Moreover, there was a positive correlation between the surplus of reducing equivalents in the pathway and the increase in product yield (Figure S5, Supporting Information). Additionally, the products in cluster 2 can be divided into two subcategories cluster 2_1 and cluster 2_2. The products in cluster 2_1 primarily involved utilizing acetyl‐CoA and its downstream metabolites as precursors and the precursors for products in cluster 2_2 were diverse (Figure 4c). Hence, for products with synthesis pathways exhibiting a surplus of reducing equivalents, strategies S1 to S4 can be chosen, and if the product synthesis pathway uses acetyl‐CoA and its downstream metabolites as precursors, strategies S5 to S7 can be chosen. For example, in the synthesis of L‐serine from glucose in *E. coli*, a total of 40 mol of NADH is generated per 10 mol of glucose. Of this, 20 mol of NADH is theoretically converted to NADPH via transhydrogenase, resulting in a surplus of 20 mol of NADH (Supporting Information, Figure S6a, Supporting Information). Since the synthesis of L‐serine has a surplus of reducing equivalents, strategies S1‐S4 can be selected to enhance its yield. However, as the precursor for L‐serine, 3PG (3‐Phospho‐D‐glycerate), is an upstream metabolite of acetyl‐CoA, strategies S5 to S7 do not improve the yield of L‐serine. For products without a surplus of reducing equivalents, selecting strategies S8 to S13 is preferable to achieve higher *Y^P^*. The synthesis of L‐asparagine from glucose in *E. coli* does not result in a surplus of reducing equivalents. The synthesis of L‐asparagine requires a large amount of ATP, and thus, a portion of the reducing equivalents (NADH, Q8H2) generated in the L‐asparagine synthesis pathway needs to be converted into ATP via the respiratory chain (Supporting Information, Figure S6b, Supporting Information). Due to the lack of surplus‐reducing equivalents in the synthesis of L‐asparagine, carbon‐conserving strategies are ineffective in improving its yield. However, the energy‐conserving strategy S8 can be employed to enhance its yield.

**Figure 5:**
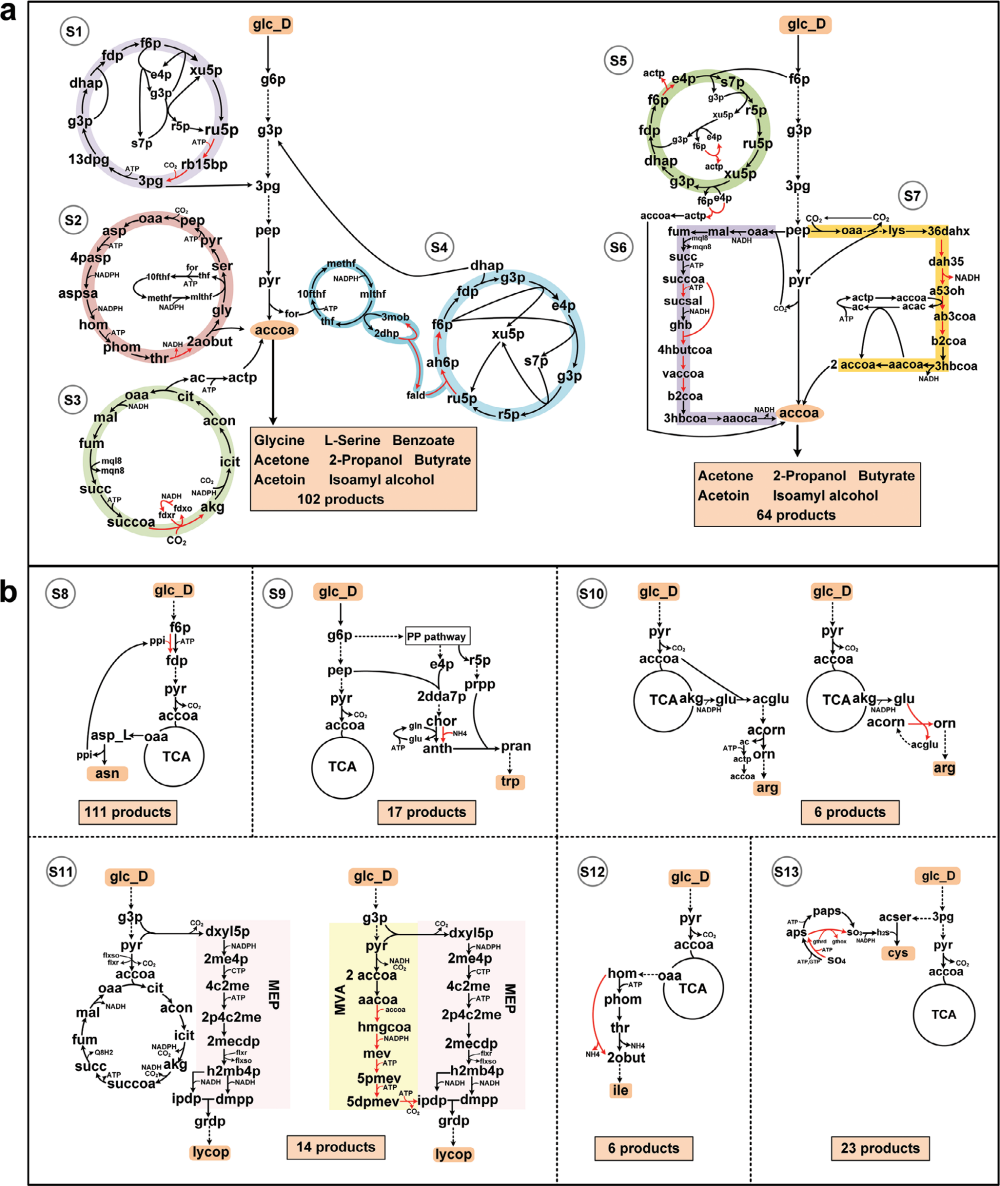
Product synthesis pathways after introducing 13 strategies. a) Product synthesis pathways after the introduction of carbon‐conserving strategies (S1–S7) in E. coli. b) Product synthesis pathways after the introduction of energy‐conserving strategies (S8–S13) in *E. coli*. The numbers in the boxes represent the number of products with increased yields after the introduction of pathway optimization strategies in *E. coli*. Red arrows indicate heterologous reactions introduced to *E. coli*. The full names of metabolite abbreviations are provided in Note S3 (Supporting Information).

To further analyze the specific mechanisms behind the improvement of *Y^P^* for each strategy, a detailed pathway analysis for each strategy was conducted. It was found that strategies S1 to S7 (cluster 2) are carbon‐conserving, primarily improving product yields by reducing carbon losses in the pathways, especially during pyruvate decarboxylation to generate CO_2_ (**Figure** **5**a). This further demonstrated that strategies S1 to S7 can utilize the surplus of reducing equivalents in the product synthetic pathways to fix carbon, thereby enhancing *Y^P^*. Strategies S1, S3, S4, and S6 are natural carbon fixation pathways,^[^ ^33^ ^]^ while strategy S2 is the threonine bypass,^[^ ^8c^ ^]^ and S5 is the non‐oxidative glycolysis pathway (NOG).^[^ ^9^ ^]^ Strategies S7 was not previously reported and produced acetyl‐CoA through the lysine derivative pathway, which recycled CO_2_ from the lysine synthesis pathway and avoided carbon loss from pyruvate to acetyl‐CoA. Moreover, these strategies were effective in enhancing *Y^P^* under various oxygen conditions (Table 1) (Tables S10–S15, Supporting Information Data 1). Among them, four carbon‐conserving strategies (S1, S2, S3, and S5) have been experimentally validated in product synthetic enhancement.^[^ ^8^, ^34^ ^]^

In contrast to the carbon‐conserving strategies, strategies S8 to S13 (cluster 1) are energy‐conserving, enhancing the energy efficiency of the synthesis pathways by reducing ATP consumption (Figure 5b). Strategy S8 introduces the heterologous reaction (PFK_ppi: f6p + ppi = > fdp + h + pi) to reduce ATP consumption, effectively enhancing the *Y^P^* of 111 products, serving as a promising general strategy. Strategy S10, introducing the reaction (ORNTAC: acorn + glu__L <=> acglu + orn), saves energy and efficiently improved the *Y^P^* of ornithine‐derived products. The *Y^P^* of L‐arginine can be increased by introducing strategy S10, which has been verified by the literature.^[^ ^35^ ^]^ Strategy S11 involves the introduction of the MVA pathway to enhance the *Y^P^* of terpenes. The MEP pathway is the native pathway in *E. coli*. The MEP pathway (0.097 mol mol^−1^ glucose) has a higher theoretical yield than the MVA pathway (0.083 mol mol^−1^ glucose). After introducing the MVA pathway, the yield of the combined MEP and MVA pathway (0.1 mol mol^−1^ glucose) is higher than both the individual MEP and MVA pathways in *E. coli*. Therefore, the synergistic action of the MVA and MEP pathways more effectively improves yields. This strategy has also been verified that the *Y^P^* of lycopene was improved by the introduction of the MVA pathway in *E. coli*.^[^ ^36^ ^]^ Other energy‐conserving strategies introduced one or two heterologous reactions to save energy (details in Note S2, Supporting Information). Interestingly, although the impact of energy‐conserving strategies on the improvement of *Y^P^* was less evident under aerobic conditions, these energy‐conserving strategies were highly effective in enhancing *Y^P^* under anaerobic or microaerobic conditions with the oxygen uptake rate setting at 2 mmol gDCW^−1^h^−1^ (Table 1). This is primarily due to the energy deficiency within cells under anaerobic or microaerobic conditions. Therefore, energy‐conserving strategies offer a new perspective to break the yield limit in a host to produce high‐value products through anaerobic fermentation.

### 2.5 Validation of the Calculated Pathways by QHEPath

To help biologists apply the model and algorithm developed in this work for quantitative heterologous pathway design, we have developed a web tool, QHEPath, accessible at https://qhepath.biodesign.ac.cn/. The primary function of QHEPath is to predict heterologous pathways that improve product yield (see Note S7, Supporting Information, for a detailed function description). To validate the pathways predicted by QHEPath, we have conducted a thorough literature search and identified several studies that used one of the heterologous pathways calculated by QHEPath to improve product yield. As shown in **Table** **2**, we summarized the calculated pathways and their experimental validation reported in the literature for six products. Through the hyperlink in the table, the precalculated pathways for the specific products and hosts can be accessed. Furthermore, the pathways experimentally validated in the literature were also visualized and compared with the pathway maps extracted from the corresponding papers (Figures S10–S15, Supporting Information).

**Table 2:** QHEPath calculated pathways that are in agreement with previously reported metabolic engineering experimental results.

Product	Host	Hyperlink to QHEPath Results	Count of Pathways	Count of Heterologous Reactions	Validated Path ID^a)^	Validated Strategy	References
Acetone	*E. coli*	https://qhepath.biodesign.ac.cn/calc‐detail/7c5c0fff‐35a6‐4b91‐a7b5‐33a223e9aa4e	12	1–9	Path 3 (Figure S10, Supporting Information)	S5	[34a]
3‐hydroxypropanoate	*E. coli*	https://qhepath.biodesign.ac.cn/calc‐detail/b2b0c588‐0d26‐4a13‐87e9‐5b2bf22f76b4	11	0–9	Path 2 (Figure S11, Supporting Information)	/	[37]
Poly(3‐hydroxybutyrate)	*E. coli*	https://qhepath.biodesign.ac.cn/calc‐detail/7c40c0da‐a600‐44f9‐b388‐4c17fdda6c0d	12	2–10	Path 4 (Figure S12, Supporting Information)	S5	[8b]
L‐arginine	*E. coli*	https://qhepath.biodesign.ac.cn/calc-detail/2daf7b11‐ee4b‐45c2‐9297‐67369f564c8e	4	0–6	Path 2 (Figure S13, Supporting Information)	S10	[35]
Spermidine	*Yeast*	https://qhepath.biodesign.ac.cn/calc-detail/ce4d0db8-753d-4651-bbef-2593eb5ff424	4	0–2	Path 3 (Figure S14, Supporting Information)	/	[23b]
Farnesen*e*	*Yeast*	https://qhepath.biodesign.ac.cn/calc‐detail/bbec3f0f‐013b‐4045‐be82‐861822705cbc	15	0–18	Path 9 (Figure S15, Supporting Information)	S5	[8a]

^a)^ The numerical identifier after “Path” is derived from the Path ID assigned by QHEPath, which can be directly accessed and visualized through the hyperlink in Table 2.

**Figure 6:**
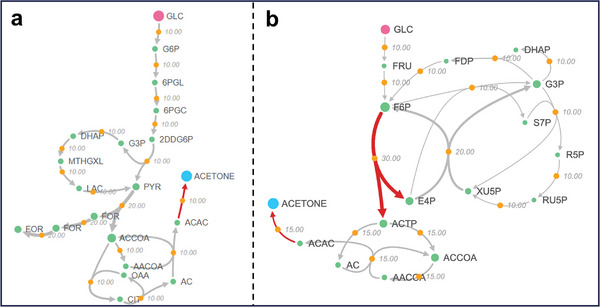
The acetone synthesis pathways predicted by QHEPath. a) The initial synthesis pathway of acetone by introducing one heterologous reaction (red lines). b) The high‐yield pathway of acetone involves the introduction of two heterologous reactions (red lines) and has been experimentally validated.

For example, Yang et al.^[^ ^34a^ ^]^ introduced the heterologous gene *fxpk*, encoding phosphoketolase (catalyzing reaction PKETF: f6p + pi = > actp + e4p + h2o), into *E. coli* to improve the acetone yield from 0.38 to 0.47 mol mol^−1^ glucose. The acetone yield of the initial pathway predicted by QHEPath in *E. coli* is low (≈ 1.0 mol mol^−1^ glucose) due to the carbon loss during the synthesis of acetyl‐CoA from pyruvate (**Figure** **6**a). QHEPath calculated several pathways that can improve the acetone yield with 1 to 9 heterologous reactions. Notably, five pathways can increase the yield from 1.0 to 1.5 mol mol^−1^ glucose. Among them, the minimum number of heterologous reactions required is just one, namely the reaction PKETF, which was used by Yang et al. (Figure 6b). The detailed description of QHEPath predicted and experimentally verified results for all products in Table 2 can be found in Note S6 (Supporting Information). These results indicate that the heterologous pathways found by QHEPath are effective engineering strategies to improve product yield. Furthermore, QHEPath calculated more potential pathways that can be experimentally tested to improve product yield.

## 3 Discussion

In cell factory construction, the yield of product synthesis is limited by the metabolic stoichiometry of the host. While metabolic engineering modifications, such as gene deletion, over‐expression, or attenuation, can enhance experimental yield, they cannot break the yield limit *Y* ^*P*0^ in a host. Breaking the limitation can only be achieved by expanding the host metabolic network through the introduction of heterologous pathway genes. Although literature reports indicate that introducing heterologous genes can break the yield limit of products, this approach has not been widely adopted in cell factory construction. Before this study, uncertainties remained regarding the feasibility of introducing heterologous reactions to increase yield for a wide range of products, as well as the existence of universal strategies for enhancing product yields. Furthermore, no available methods addressed this gap. Our work introduces a computational workflow to systematically tackle this issue. The results of our study are highly encouraging as they show that introducing heterologous reactions is broadly effective for 70% of the products to break the yield limit *Y* ^*P*0^ in hosts. Moreover, we systematically summarized multiple engineering strategies and their applicable product characteristics, including the surplus of reducing equivalents and synthesis precursors, providing a new perspective on metabolic pathway optimization. An online web tool for quantitative heterologous pathway design has been developed, enabling biologists to conveniently utilize the model CSMN and the algorithm for pathway design and optimization.

Our quality‐control workflow ensures the preservation of more reactions from the BiGG database. The principle of the error elimination algorithm is to minimize changes to the reactions while ensuring the correct calculation of product pathways. Unbalanced reactions were penalized, but not all received the highest penalty. In the penalty rules, unbalanced reactions were categorized into two types: those with carbon unbalances, which were penalized with the highest score of 3, and those involving H or H_2_O, which were not penalized. This distinction is because H or H_2_O molecules can be balanced through exchange reactions and therefore do not affect the calculation of product pathways. Consequently, this algorithm allows for the retention of some unbalanced reactions that do not affect the calculation of product pathways, thereby maintaining the diversity of reactions. In contrast to the universal model constructed by CarveMe,^[^ ^38^ ^]^ CSMN included almost all reactions from the BiGG database and these reactions were curated through an automated quality‐control workflow, while CarveMe curated errors for only 15% of (4383/28301) reactions from the BiGG. This comprehensive coverage provides the potential for optimizing pathways for a broader range of products. For example, CSMN can compute pathways for all 300 products, while CarveMe can only calculate pathways for 55% (166 out of 300) of the products (Supporting Data 3). Moreover, in the event of potential updates to the BiGG database in the future, the CSMN can be seamlessly and automatically updated through the established quality‐control workflow.

The development of QHEPath was inspired by the minimal heterologous reaction concept from OptStrain^[^ ^17^ ^]^ Unlike OptStrain, the QHEPath can identify the heterologous reactions contributing to the improvement of *Y^P^* for non‐native products. Moreover, it can calculate multiple suboptimal and optimal pathways, which helps select a product pathway with fewer heterologous reactions while maintaining *Y^P^* close to $Y_{m}^{P}$ for metabolic engineering modifications. We have tested 300 products from diverse categories to demonstrate the effectiveness of introducing heterologous pathways through the application of this algorithm. However, the *Y^P^* of some products did not reach *Y^E^*, which implied potential space for further optimization. One approach to address this limitation is to enhance the diversity of reactions within the CSMN model by incorporating non‐natural reactions. Recently, Homa et al.^[^ ^39^ ^]^ developed the comprehensive biochemical database ATLASx, which includes over five million predicted reactions based on generalized enzymatic reaction rules. The inclusion of this extensive resource significantly expands the pool of available reactions for pathway optimization, offering valuable insights for future research.

Additionally, the product pathways predicted are likely to show higher yields than those observed experimentally due to many factors, such as enzyme kinetics, metabolic burden, and the accumulation of toxic intermediates.^[^ ^40^ ^]^ To bring experimental yields closer to the predicted theoretical yields, combining heterologous introduction strategies with other metabolic engineering strategies (screening/engineering enzymes to improve enzyme activity, deleting genes to reduce byproducts, and rewiring the regulation system to balance metabolism) holds promise for further enhancing yields.^[^ ^41^ ^]^ While pathway design is just one of the steps in constructing strains, it serves as the starting and focal point for subsequent enzyme and metabolic engineering research.

In summary, our results underline the great potential of enhancing pathway yield by introducing heterologous pathways for the rational engineering of cell factories. We have demonstrated that such designs are, in principle, widely realizable in all production organisms investigated. QHEPath has successfully predicted biologically plausible pathways validated in literature for multiple products.^[^ ^8^, ^23^, ^24^, ^34^, ^35^, ^37^, ^42^ ^]^ We expect that our research will facilitate the more successful construction of efficient product pathways in the future.

## 4 Experimental Section

### Model Preprocessing

The universal model (version 1.6) was downloaded from the BiGG database (http://bigg.ucsd.edu/), which contains reactions from the 108 genome‐scale metabolic models (GEMs) and was used as the initial model. The charge and formula of the metabolites were extracted from the 108 GEMs. Reaction directions were initially determined based on the maximum count among the three directions (forward, backward, and reversible) in the GEMs. Subsequently, reactions that appeared in fewer than ten GEMs, or had a reaction direction frequency (F) lower than 0.7 (Equation 1), were subjected to thermodynamic assessment using eQuilibrator 3.0.^[^ ^43^ ^]^ This analysis was performed to correct the reaction directions and ensure the accuracy of the metabolic network. We set the metabolite concentration between 0.01 and 10mm. ^[^ ^44^ ^]^ Reactions were classified as irreversible if their upper bound of the Gibbs free energy was less than 0 kJ mol^−1^ or their lower bound was greater than 0 kJ mol^−1^. For reactions without the Gibbs free energy, we used heuristic rules to determine reaction directions^[^ ^20^ ^]^: i) for most reactions containing oxygen, their directions were assumed to be that of the oxygen consumption; ii) most reactions produced NH_3_/NH_4_^+^, except when it reacted with ATP, 2‐oxoglutarate, chorismate, 5‐phospho‐alpha‐D‐ribose‐1‐diphosphate, and UDP‐N‐acetyl‐beta‐L‐fucosamine; iii) most reactions cannot proceed in the direction of CO_2_ fixation, except for naturally known carbon fixation reactions^[^ ^45^ ^]^ and reactions utilizing CO_2_ and high‐energy substrates such as phosphoenolpyruvate (PEP) and ATP; iv) most reactions involving ATP, GTP, ITP, UTP are in the consumption direction except for reactions with another high energy metabolite such as Acyl‐CoA.

The frequency of reaction directions, denoted as F, was calculated using the following Equation (1):

(1) $$F=\frac{\max\left(N_{f},N_{b},N_{r}\right)}{N_{f}+N_{b}+N_{r}}$$

Here, *N_f_*, *N_b_*, *N_r_* represented the counts of the same reaction appearing in different GEMs in the forward, backward, and reversible directions, respectively.

### Calculating the Maximum Theoretical Yield of a Product

The maximum theoretical yield of the product (*Y^E^*) represents the maximum amount of a product that can be formed from a substrate and is calculated solely based on the reduction degrees of the substrate and the product^[^ ^7^ ^]^ (Equation 2). It is pathway‐independent and provides yield limits for the product pathway, irrespective of the process or catalyst used.

(2) $$Y^{E}=\frac{Rd_{s}}{Rd_{p}}$$

Here, *Rd_s_* is the reduction degree of the substrate, and *Rd_p_* is the reduction degree of the product. For instance, the reduction degree of lactate (*Rd_p_*) is calculated as C_3_H_6_O_3_ 3×4+6×1+3×(−2) = 12, while that of glucose (*Rd_s_*) is calculated as C_6_H_12_O_6_ 6×4+12×1+6×(−2) = 24. Therefore, *Y^E^* of lactate is 2 (24/12) mol mol^−1^ glucose.

### Eliminating Different Types of Errors within the Universal Model

The error elimination method was developed to identify and correct errors including infinite generation of metabolites, reducing equivalents, and energy, as well as incorrect pathways with yields exceeding *Y^E^* in the model. The workflow of error elimination methods is illustrated in Figure 1. This algorithm was developed based on iterative pFBA calculations and consists of three main parts. Initially, it penalizes untrusted reactions that include charge and mass unbalanced reactions and unreliable reaction directions. The penalty rules are as follows: i) reactions with inconsistencies in charge and mass, except for exchange and transport reactions, were fined three points; ii) reactions without annotation information that represented external database links were fined one point; iii) reactions appearing in less than 10 GEMs were fined one point. Penalty values were derived based on the error severity of incorrect reactions by establishing penalty rules. In the penalty rules, reactions with inconsistencies in charge and mass received the highest penalty of three points, as such errors are most likely to cause significant model inaccuracies. Reactions lacking annotation information or appearing in fewer than 10 GEMs were each penalized one point, as these errors are less likely to severely impact model accuracy. These penalty values are not strict scores but rather indicators of error severity, helping to prioritize reactions most likely to introduce significant model errors.

Subsequently, the objective function and threshold were set for different error types in the pFBA calculations. If the threshold was not satisfied, reactions with the highest score in the pFBA result were removed. The pFBA calculation was performed iteratively until the constraint was satisfied, leading to the generation of Model^R^ that was free of corresponding errors. Simultaneously, all removed reactions were appended in set^removed^. Finally, reactions in set^removed^ were added back to Model^R^ one by one, and pFBA was performed. If the threshold was satisfied after restoring a reaction, it was returned to Model^R^. Conversely, if the above threshold was not satisfied after restoring the reaction, it was deemed to have caused the corresponding error and was either removed (in the case of charge and mass unbalances) or corrected (in the case of incorrect reaction direction).

After locating these error‐causing reactions, they were handled and corrected according to the following rules. 1) The error‐causing reactions, which are mass‐unbalanced and belong to the categories of coefficient errors, missing cofactors, or incorrect metabolites, were corrected by comparing them with mass‐balanced reactions. 2) The error‐causing reactions, which are mass‐unbalanced and belong to macromolecule synthesis or biomass reactions, or lack annotation information, were directly removed. 3) The error‐causing reactions, which are mass‐balanced, were corrected by closing the error‐causing direction and retaining the correct direction.

The error elimination algorithm was employed to identify the reactions quickly and accurately leading to different types of errors in this model. For the infinite generation of metabolites, demand reactions (DM_metabolite: metabolite_c = >) for metabolites containing carbon and other element (oxygen, nitrogen, phosphorus, sulfur) were added to the model and set as an objective function iteratively to check the infinite production of these elements. The threshold was that the flux of the reaction DM_metabolite was zero, which ensured that the metabolite was no longer generated indefinitely. For the infinite generation of reducing equivalents, demand reactions (DM_NADH: nadh_c = > nad_c + h_c) of five metabolites (NADH, NADPH, Flavin adenine dinucleotide, Ubiquinol‐8, 2‐Demethylmenaquinol 8), were added into the model and set as the objective function. The threshold was that the flux of each demand reaction was zero without carbon source supply.

In terms of infinite generation of energy, respiratory chain reactions formed the proton gradient to produce energy.^[^ ^16^ ^]^ To avoid a variety of respiratory chains in 108 GEMs causing wrong energy generation, 21 respiratory chain reactions were deleted and combined respiratory chain reactions were added to make ATP production and proton gradient unrelated.^[^ ^21b^ ^]^ Moreover, the dissipation reactions (ATPM: atp_c + h2o_c = > adp_c + pi_c + h_c) of energy metabolites (ATP, CTP, GTP, UTP, ITP) were set as objective functions respectively, and the threshold was that the flux of the dissipation reaction of energy metabolite ATPM was zero without carbon source supply. Metabolites with incorrect synthetic pathways indicate that the pathway yield exceeded *Y^E^* in the model. The error elimination algorithm can also be used to locate reactions causing this type of error. Demand reactions for metabolites with pathway yields exceeding *Y^E^* (DM_metabolite: metabolite_c = >) were added to the model and set as the objective function. The threshold was that the flux of the reaction (DM_ metabolite) did not exceed *Y^E^* by supplying carbon source glucose.

### Algorithm for Quantitative Heterologous Pathway Design

To directly distinguish heterologous reactions responsible for enhancing *Y^P^* and calculate multiple suboptimal and optimal pathways, we have developed a quantitative heterologous pathway design algorithm. This algorithm mainly consisted of four steps. In Step 1, the maximum flux of the product pathway ($v_{m}^{P}$) was calculated in the model CSMN by performing FBA.^[^ ^11a^ ^]^

Step 2, calculating the minimum number of heterologous reactions (N_syn_) required to achieve the producibility of non‐native products (Equations (3), (4), (5), (6)).

(3) $$\min\sum_{j\in R_{\mathrm{hetr}}}y_{j}$$

(4) $$S_{ij}\cdot \;v_{j}=\;0$$

(5) $$v_{j}^{lb}\cdot y_{j}\leq \;v_{j}\leq v_{j}^{ub}\cdot y_{j}$$

(6) $$0.1v_{m}^{P}\leq v_{product}$$

where the set *R_hetr_* represented heterologous reactions when specified in a host organism. *y_j_* denoted the binary variable. *v_product_* was the flux of the target product. *S_ij_* represented the stoichiometric matrix of the model CSMN and *v_j_* was the flux of reactions in model CSMN. $v_{j}^{lb}$ and $v_{j}^{ub}$ represented the lower bound and upper bound of reaction flux, respectively. Equation (3) was to minimize the number of heterologous reactions. Equation (4) describes the steady‐state mass balance constraints determined by the stoichiometric matrix. Equation (5) showed whether the heterologous reaction was introduced into the chassis. Equation (6) indicates the flux of the target product (*v_product_*) was not less than 10% of $v_{m}^{P}$.

Step 3, calculating the minimum number of heterologous reactions (N_opt_) required to achieve the $v_{m}^{P}$ (Equations (7), (8), (9), (10)).

(7) $$\min\sum_{j\in R_{\mathrm{hetr}}}y_{j}$$

(8) $$S_{ij}\cdot \;v_{j}=\;0$$

(9) $$v_{j}^{lb}\cdot y_{j}\leq \;v_{j}\leq v_{j}^{ub}\cdot y_{j}$$

(10) $$v_{product}=\;v_{m}^{P}$$

Here, Equation (7) was to minimize the number of heterologous reactions. Equation (10) indicated that the flux of the target product (*v_product_*) reached $v_{m}^{P}$.

Step 4, the stepwise introduction of heterologous reactions from N_syn_+1 to N_opt_‐1 to demonstrate the optimization process and obtain multiple optimization pathways (Equation (11), (12), (13), (14), (15)).

(11) $$\max\;v_{product}$$

(12) $$S_{ij}\cdot \;v_{j}=\;0$$

(13) $$v_{j}^{lb}\cdot y_{j}\leq \;v_{j}\leq v_{j}^{ub}\cdot y_{j}$$

(14) $$0.1v_{m}^{P}<v_{product}<\;v_{m}^{P}$$

(15) $$\sum_{j\in R_{\mathrm{hetr}}}\;y_{j}\;\in N\;\left(N=N_{\mathrm{syn}}\;+1,N_{\min}+2,\cdots N_{\mathrm{opt}}-1\right)$$

Here, Equation (11) represented the maximization of the target product flux (*v_product_*). Concurrently, *v_product_* was not surpass $v_{m}^{P}$ (Equation 14). Equation (15) showed heterologous reactions were introduced stepwise, and the range of the number of heterologous reactions was from the N_syn_ +1 to N_opt_ −1

### Calculating the Surplus of Reducing Equivalents of a Pathway

To identify the characteristics of the products in the cluster analysis, we calculated the surplus of reducing equivalents of a pathway using *E. coli* as the chassis. First, we set the boundary of the demand reaction for the specific product to its optimal rate, considering a glucose uptake rate of 10 mmol gDCW^−1^h^−1^. Next, we introduced the reaction (ADD_NADH: nadh_c + h_c = > nad_c) into the chassis model and utilized it as the objective function for performing pFBA calculations. The flux of the reaction ADD_NADH represented the surplus reducing of equivalents in the product pathway. For example, the surplus reducing of equivalents in the synthesis pathway of acetone was 40 mmol per 10 mmol of glucose.

### Calculating the Precursor of Product Synthesis

To identify the characteristics of the products in the cluster analysis, the precursor compounds for product synthesis were calculated using *E. coli* as the chassis by the following calculation method. Sink reactions were added for the 12 precursor compounds (e4p, pep, r5p, oaa, 3pg, pyr, f6p, g6p, g3p, accoa, akg, succ) in the central carbon metabolism to ensure an unlimited supply of these compounds. Reactions to supply reducing equivalents (ADD_NADH: nadh_c + h_c <=> nad_c, ADD_NADPH: nadph_c + h_c <=> nadp_c) were introduced, and the ATP maintenance reaction (ATPM: atp_c + h2o_c <=> adp_c + h_c + pi_c) was set as reversible to provide the infinite supply of reducing equivalents and energy. The demand reaction for the product was set as the objective function, and pFBA calculation was performed to determine the synthesis pathway. The precursor compounds were determined by examining the sink reactions of precursor compounds to determine if they carry flux. For example, the precursor compounds for the synthesis of L‐tryptophan were identified as e4p, pep, and r5p.

### Website Construction

To facilitate quantitative heterologous pathway design for biologists using the CSMN model and the algorithm, we built a web tool, QHEPath. The website was built on AWS S3, enabling static website hosting and AWS CloudFront to boost speed. AWS Fargate was utilized to execute the compute script, while AWS API Gateway served as the API server, efficiently handling HTTP requests and routing them to the appropriate backends. AWS Step Functions were employed to facilitate message processing and invoked AWS Fargate in an asynchronous manner. Finally, the entire infrastructure was deployed using AWS CloudFormation, which enabled rapid and consistent provisioning while treating the infrastructure as code.

## Conflict of Interest

The authors declare no conflict of interest.

## Author Contributions

F.W. and J.C. equally contributed to this work. H.M. and Q.Y. designed and supervised the whole research. F.W., Q.Y., J.C., and Y.M. contributed concept and implementation. F.W., Y.W., H.L., and Z.M. contributed the development of the webserver. X.L., A.L., X.D., and F.L. participated in the discussion and revision of the manuscript. F.W., Q.Y., J.C., and Y.M. wrote the manuscript. All authors reviewed and approved the final manuscript.

## Supporting information

Supporting Information

Supporting Information

Supporting Information

Supporting Information

## Data Availability

The data that support the findings of this study are available in the supplementary material of this article.
