# Supplementary material for: Unveiling Metabolic Engineering Strategies by Quantitative Heterologous Pathway Design
Source: Adv Sci (Weinh). 2024 Oct 16;11(45):2404632. doi: 10.1002/advs.202404632 (PMC11615770; doi:10.1002/advs.202404632)
Supplement: Supplementary file 1 — Supporting Information [file ADVS-11-2404632-s004.docx]

**Unveiling Metabolic Engineering Strategies by Quantitative Heterologous Pathway Design**

*Fan Wei, Jingyi Cai, Yufeng Mao, Ruoyu Wang, Haoran Li, Zhitao Mao, Xiaoping Liao, Aonan Li, Xiaogui Deng, Feiran Li, Qianqian Yuan*, Hongwu Ma**


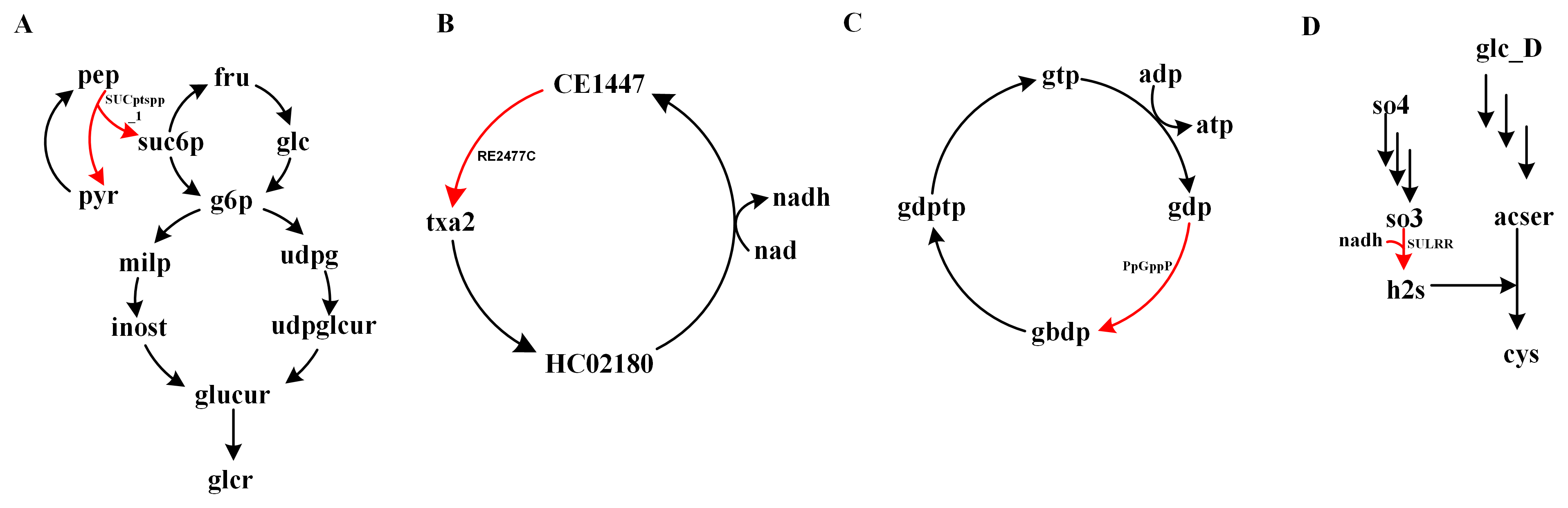


**Figure S1.** Four types of errors in the model CSMN. (A) Metabolite infinite generation. Mass unbalance reaction SUCptspp_1 caused a loop to produce glcr without consuming any substrate. (B) The infinite generation of reducing equivalents. Mass unbalance reaction RE2477C caused a loop to produce NADH. (C) Energy infinite generation. Incorrect reaction direction PpGppP caused a loop to produce ATP. (D) Incorrect pathway. Mass unbalance reaction SULRR caused the pathway yield of L-cysteine to exceed the maximum theoretical yield.


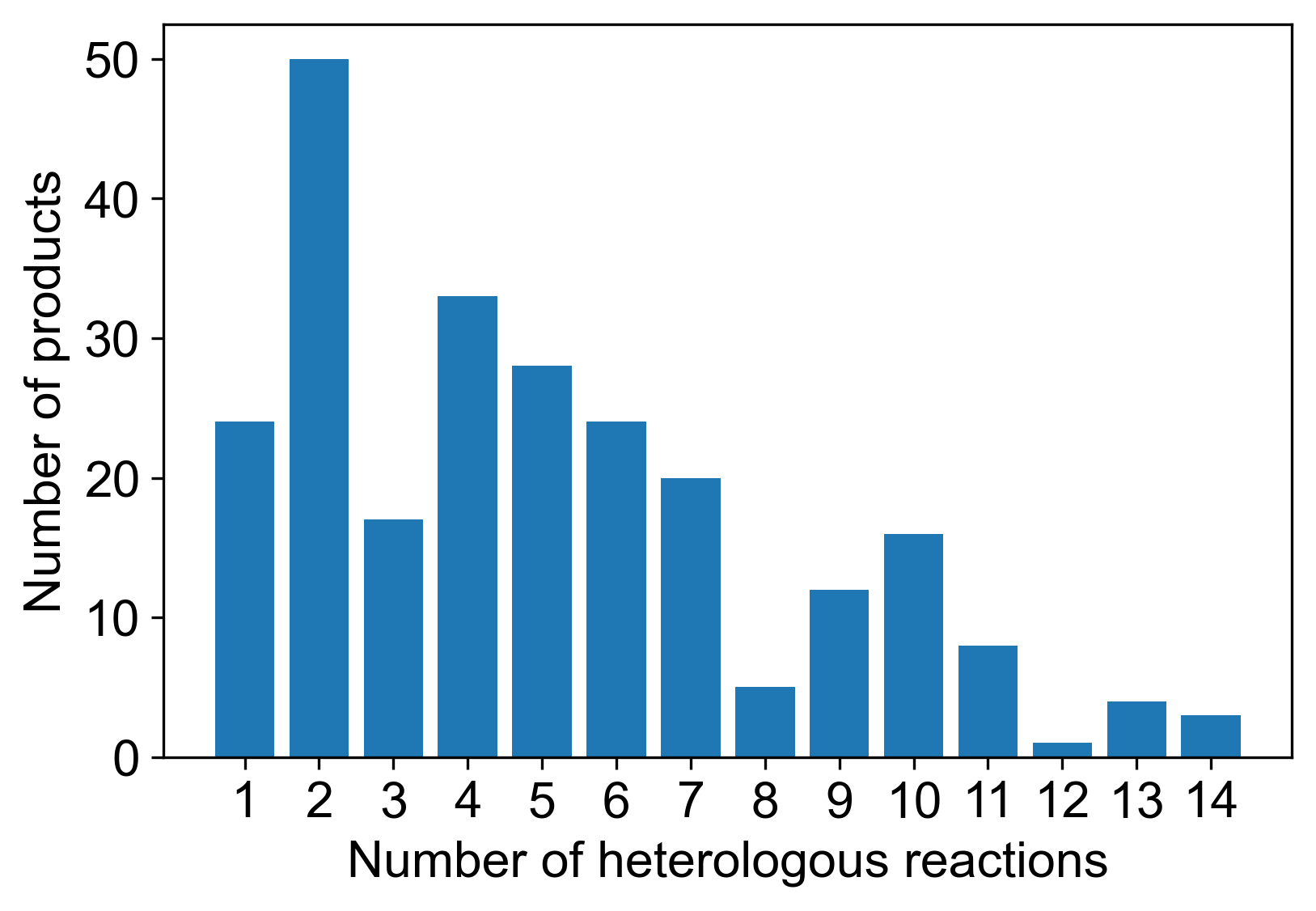


**Figure S2.** The number of products and the heterologous reactions required to enhance product yield in *E. coli* using glucose as the substrate.


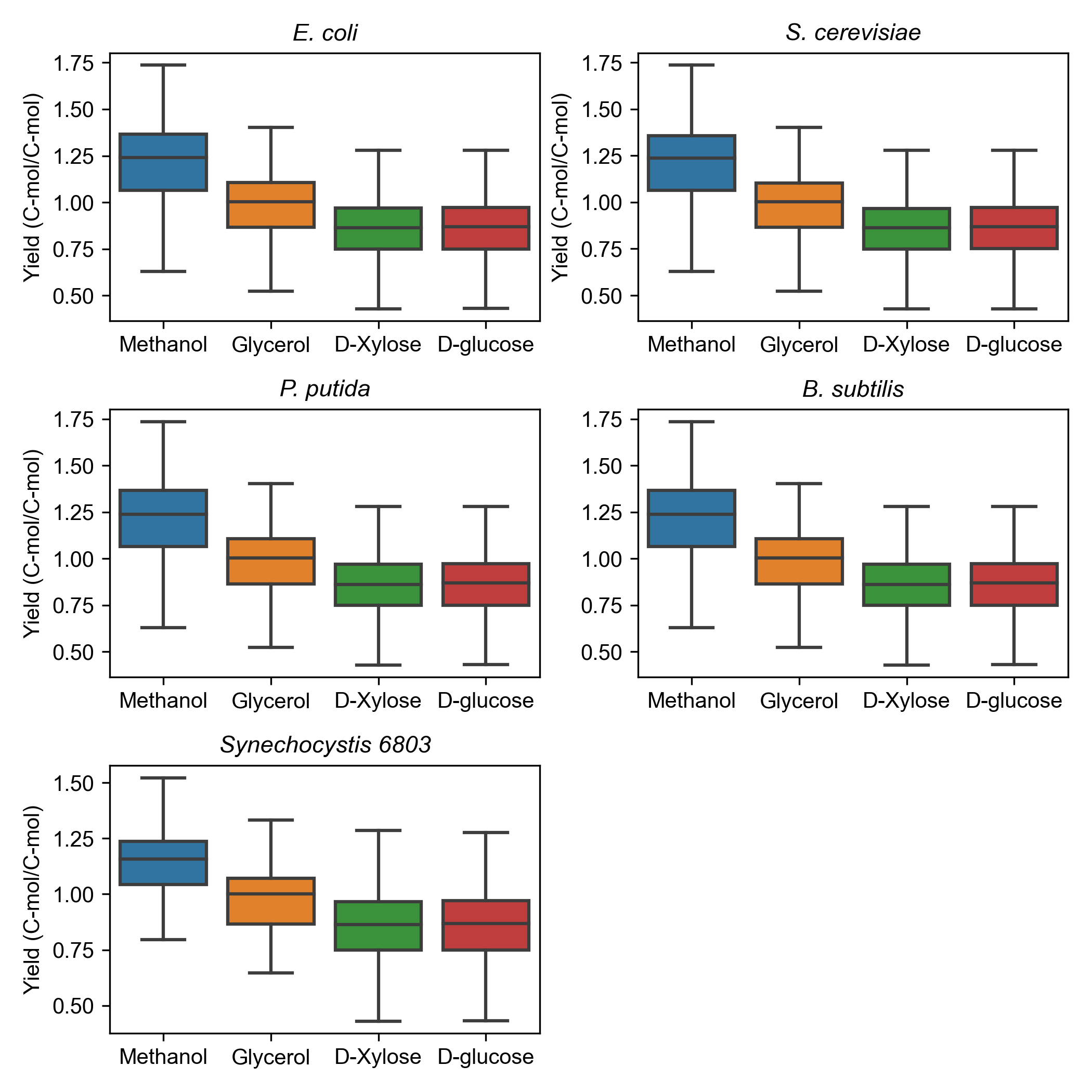


**Figure S3.** Yields of 300 products across different substrates and species.


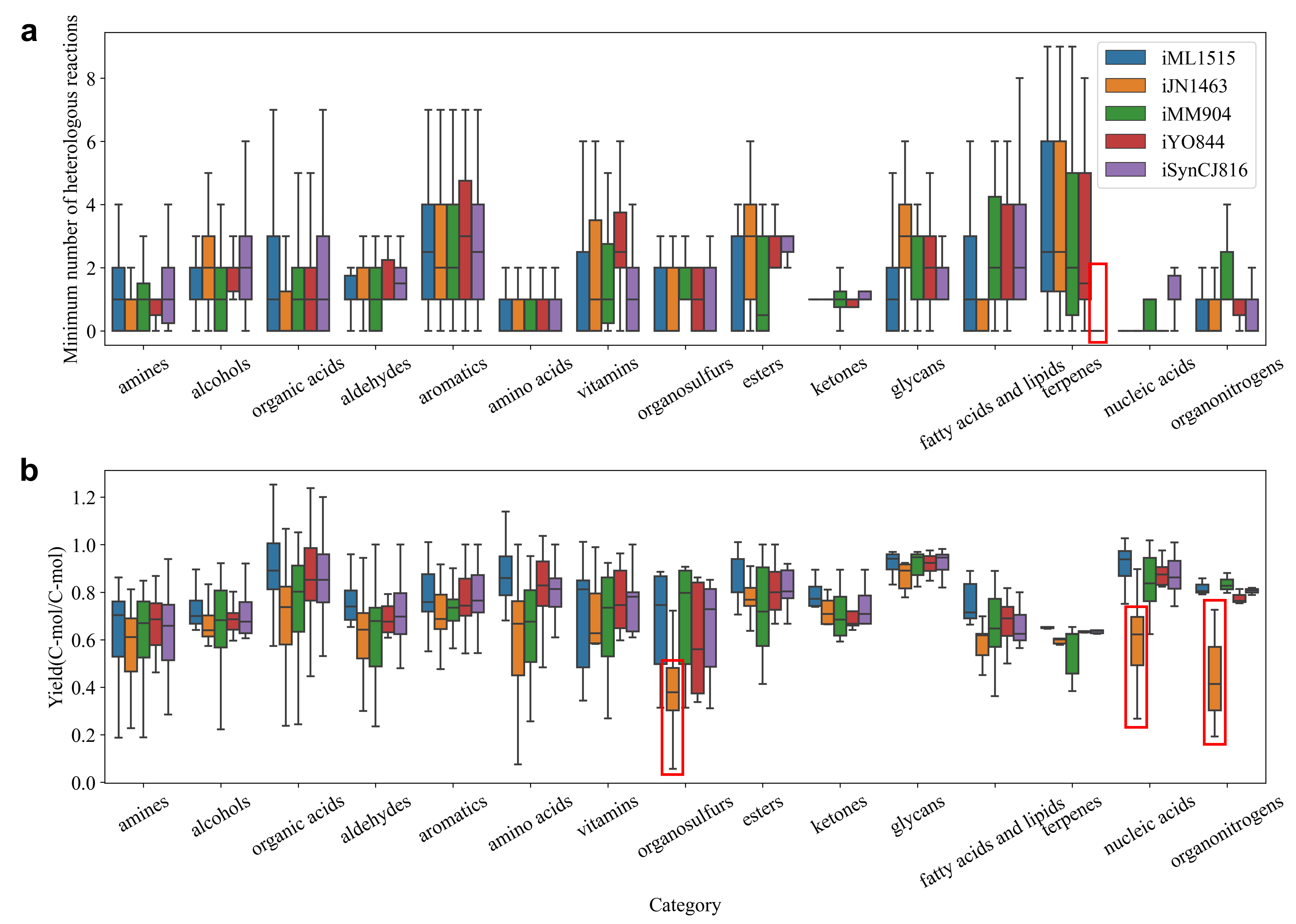


**Figure S4.** Evaluation of product synthesis in different host. (a) The minimum number of heterologous reactions required for the synthesis of 15 categories of products in five hosts. (b) The yield of 15 categories of products in five hosts.


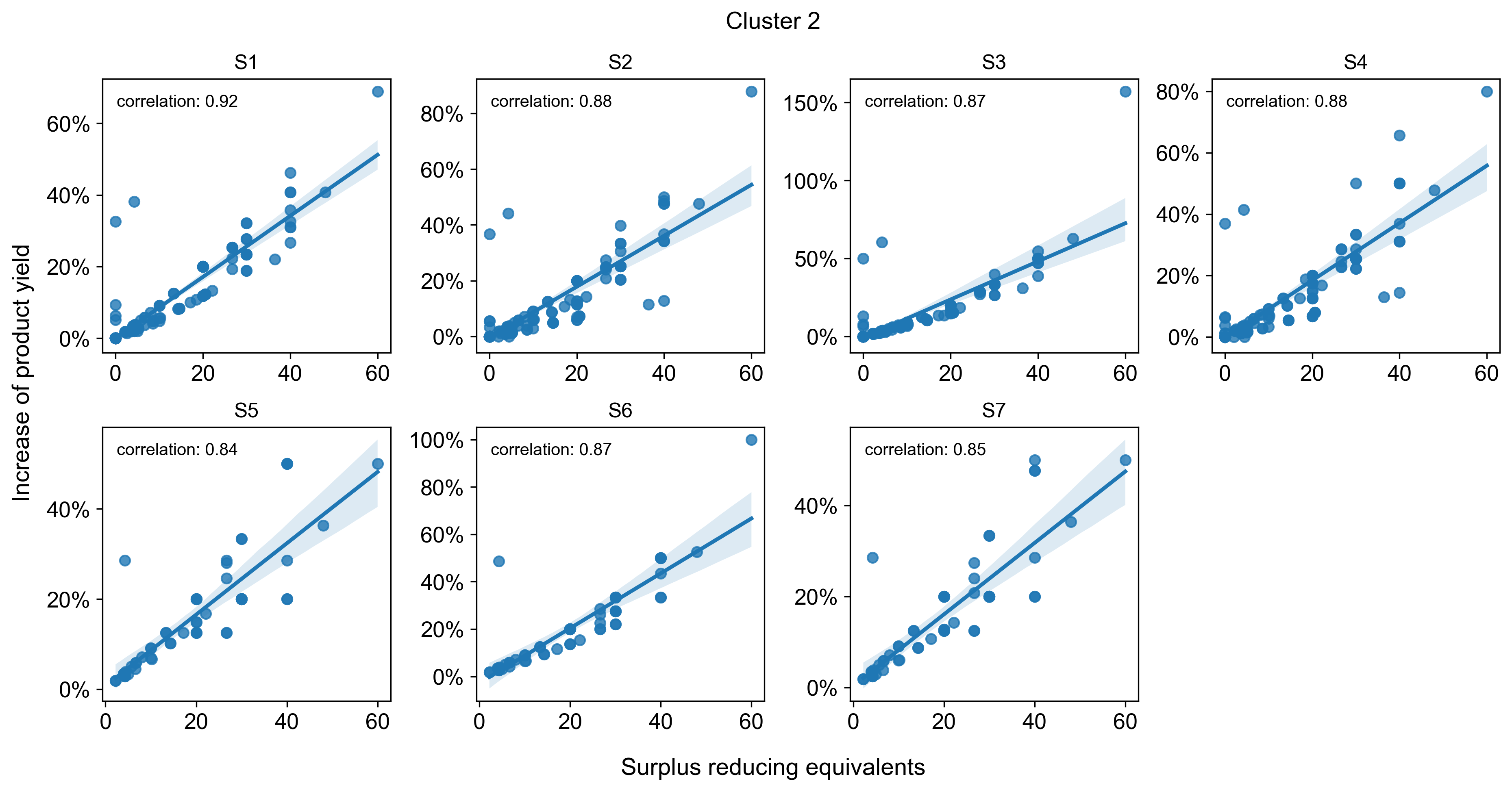


**Figure S5.** Correlation analysis between the increase of product yield and the surplus of reducing equivalents for cluster 2.


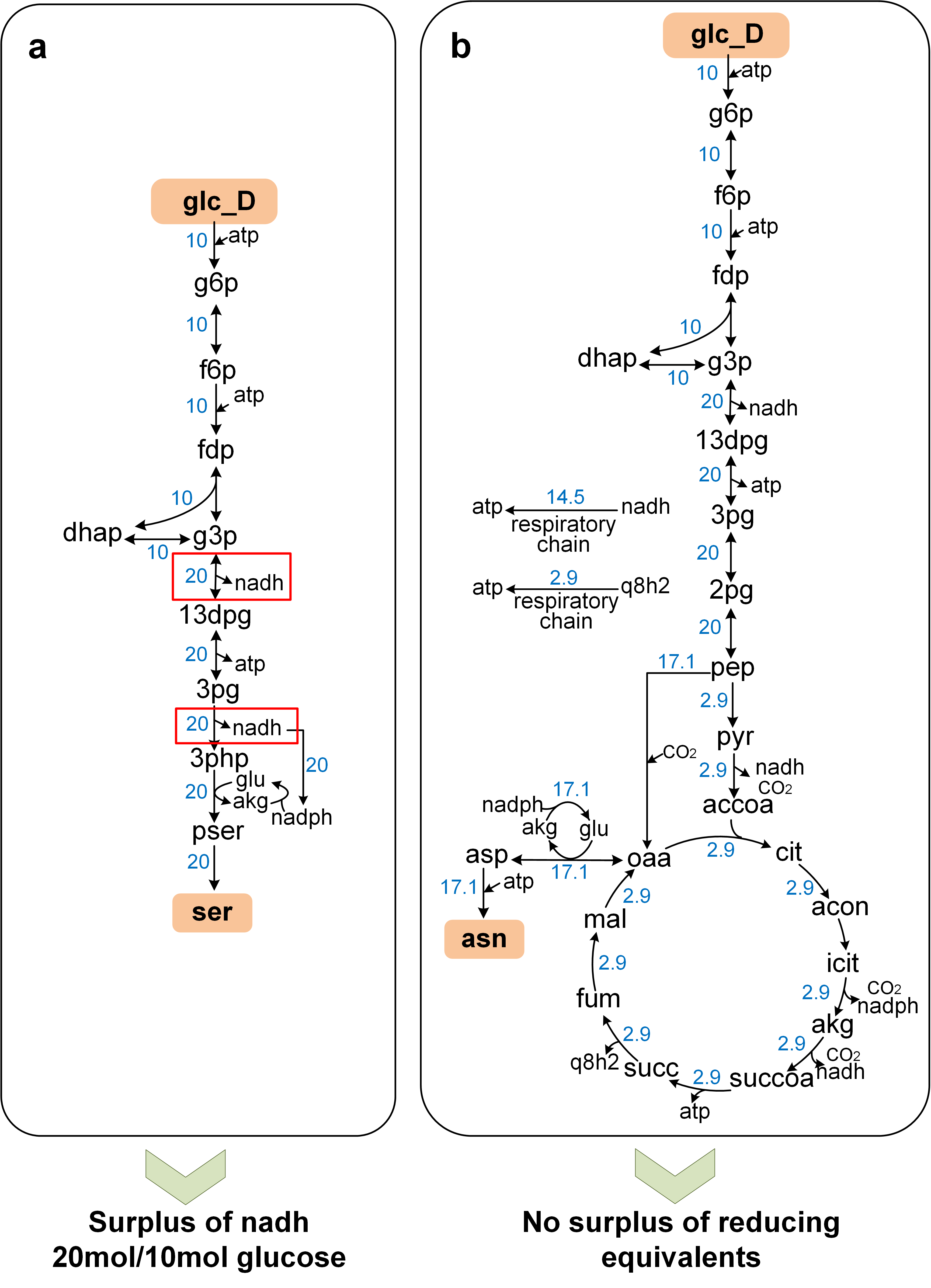


**Figure S6**. Product synthesis pathways with and without a surplus of reducing equivalents. (a) The L-serine synthesis pathway has a surplus of 20 mol of NADH from glucose in *E. coli*. (b) The L-asparagine synthesis pathway does not have a surplus of reducing equivalents from glucose in *E. coli*. glc_D, D-glucose; g6p, D-glucose 6-phosphate; f6p, D-fructose 6-phosphate; fdp, D-fructose 1,6-bisphosphate; dhap, dihydroxyacetone phosphate; g3p, glyceraldehyde 3-phosphate; 13dpg, 3-phospho-D-glyceroyl phosphate; 3pg, 3-phospho-D-glycerate; 3php, 3-phosphohydroxypyruvate; pser, O-phospho-L-serine; glu, L-glutamate; akg, 2-oxoglutarate; pyr, pyruvate; accoa, acetyl-coA; cit, citrate; acon, cis-aconitate; icit, isocitrate; succoa, succinyl-coa; succ, succinate; fum, fumarate; mal, L-malate; oaa, oxaloacetate; asn, L-asparagine; asp, L-aspartate.

**Table S1.** The percentage of the number of products with improved yields across different substrates and species under aerobic and anaerobic conditions.

|  | Methanol (%) | | Glycerol (%) | | D-Xylose (%) | | D-Glucose (%) | |
| --- | --- | --- | --- | --- | --- | --- | --- | --- |
|  | aerobic | anaerobic | aerobic | anaerobic | aerobic | anaerobic | aerobic | anaerobic |
| *E. coli* | 100 | 70 | 91.3 | 94.2 | 71 | 80.4 | 74.7 | 81.3 |
| *S. cerevisiae* | 100 | 90 | 98.3 | 96.2 | 83.7 | 93.7 | 86.3 | 95 |
| *P. putida* | 100 | / | 98 | / | 96 | / | 95.3 | / |
| *B. subtilis* | 100 | / | 100 | / | 91.9 | / | 89.9 | / |
| *Synechocystis 6803* | 100 | / | 97.3 | / | 87.2 | / | 86.9 | / |

# Note S1

**The problems with quality control algorithms**

MEMOTE^[1]^, a standardized tool for quality testing and scoring of genome-scale metabolic models (GEMs), can detect the net generation of metabolites and energy. However, it cannot pinpoint the reactions causing the errors. Furthermore, Fritzemeier *et al* ^[2]^ used the variant of the G_LOBALFIT_ algorithm to eliminate erroneous cycles of net energy generation by the removal of minimal reaction sets. Although this algorithm can find and locate the error, it cannot locate precisely the reaction that causes the error. For example, the direction of reaction ACALD (acald_c + coa_c + nad_c ⇌ accoa_c + h_c + nadh_c) in model iJN746 was modified to eliminate incorrect net energy generation cycle. However, the reaction ACALD was correct and the reverse reaction direction of the reaction ALDD2xr (acald_c + h2o_c + nad_c ⇌ ac_c + 2.0 h_c + nadh_c) should be closed.

# Note S2

**Energy-conserving strategies**

Strategies S8 to S13 were energy-conserving and they enhanced the energy efficiency of the synthesis pathways by optimizing ATP utilization. Strategy S9 introduced anthranilate synthase heterologously to convert chorismite to anthranilate with NH_+_^4^ instead of L-glutamine, resulting in ATP savings. It has been reported that anthranilate synthase can use NH_+_^4^ as the amino donor in vitro^[3]^. Strategy S12 introduced a heterologous reaction to produce 2-oxobutanoate without energy consumption, thereby replacing the native pathway that requires ATP consumption in *E. coli.* Strategy S13 introduced two heterologous reactions to convert sulfate to sulfite, consuming only one molecule of ATP instead of the high-energy consumption native pathway of *E. coli*.

# Note S3

**The full names of metabolite abbreviations**

glc_D, D-glucose; g6p, D-glucose 6-phosphate; g3p, glyceraldehyde 3-phosphate; 3pg, 3-phospho-D-glycerate; pep, phosphoenolpyruvate; pyr, pyruvate; f6p, D-fructose 6-phosphate; fdp, D-fructose 1,6-bisphosphate; dhap, dihydroxyacetone phosphate; 13dpg, 3-phospho-D-glyceroyl phosphate; xu5p, L-xylulose 5-phosphate; ru5p, L-ribulose 5-phosphate; r5p, alpha-D-Ribose 5-phosphate, s7p, sedoheptulose 7-phosphate; e4p, D-erythrose 4-phosphate; accoa, acetyl-coA; oaa, oxaloacetate; asp, L-Aspartate; 4pasp, 4-phospho-L-aspartate; aspsa, L-aspartate 4-semialdehyde; hom, L-homoserine; phom, O-phospho-L-homoserine; thr, L-threonine; 2aobut, L-2-amino-3-oxobutanoate; gly, glycine; ser, L-serine; ac, acetate; actp, acetyl phosphate; cit, citrate; acon, cis-aconitate; icit, isocitrate; akg, 2-oxoglutarate; succoa, succinyl-coa; succ, succinate; fum, fumarate; mal, L-malate; for, formate; 10fthf, 10-formyltetrahydrofolate; methf, 5,10-methenyltetrahydrofolate; mlthf, 5,10-methylenetetrahydrofolate; thf, 5,6,7,8-tetrahydrofolate; 3mob, 3-methyl-2-oxobutanoate; 2dhp, 2-dehydropantoate; fald, formaldehyde; ah6p, D-arabino-3-Hexulose 6-phosphate; actp, acetyl phosphate; sucsal, succinic semialdehyde; ghb, 4-hydroxybutanoate;4hbutcoa, 4-hydroxybutanoyl-coA; vaccoa, vinylacetyl-coA; b2coa, crotonoyl-coA; 3hbcoa, (S)-3-hydroxybutanoyl-coA; aacoa, acetoacetyl-coA; lys, L-lysine; 36dahx, (3S)-3,6-diaminohexanoate; dah35, (3S,5S)-3,5-diaminohexanoate; a53oh, (S)-5-amino-3-oxohexanoate; ab3coa, L-3-aminobutyryl-CoA; ppi, diphosphate; asn, L-asparagine; 2dda7p, 2-dehydro-3-deoxy-D-arabino-heptonate 7-phosphate; prpp, 5-phospho-alpha-D-ribose 1-diphosphate; chor, chorismite; anth, anthranilate; pran, N-(5-Phospho-D-ribosyl)anthranilate; trp, L-tryptophan; acglu, N-acetyl-L-glutamate; acorn, N2-Acetyl-L-ornithine; orn, ornithine; arg, L-arginine; dxyl5p, 1-deoxy-D-xylulose 5-phosphate; 2me4p, 2-C-methyl-D-erythritol 4-phosphate; 4c2me, 4-(cytidine 5’-diphospho)-2-C-methyl-D-erythritol; 2p4c2me, 2-phospho-4-(cytidine 5'-diphospho)-2-C-methyl-D-erythritol; 2mecdp, 2-C-methyl-D-erythritol 2,4-cyclodiphosphate; h2mb4p, 1-hydroxy-2-methyl-2-(E)-butenyl 4-diphosphate; ipdp, isopentenyl diphosphate; dmpp, dimethylallyl diphosphate; grdp, geranyl diphosphate; hmgcoa, hydroxymethylglutaryl-coA; mev, mevalonic acid; 5pmev, (R)-5-Phosphomevalonat; 5dpmev, ®-5-Diphosphomevalonate; lycop, lycopene; ile, L-isoleucine; cys, L-cysteine.

# Note S4

**Evaluation of the parameter stability and computational efficiency of the QHEPath algorithm**

To evaluate the computational efficiency of the QHEPath algorithm, we tested the computation time required to predict optimized pathways for 300 products using different substrates in *E. coli*. This evaluation was conducted on a server equipped with an AMD EPYC 7763 64-Core Processor and 512GB of RAM. We found that for most products, the computation time ranged from approximately 3 to 10 minutes (Figure S7). However, pathways with more steps required longer computation times, exceeding 19 minutes.


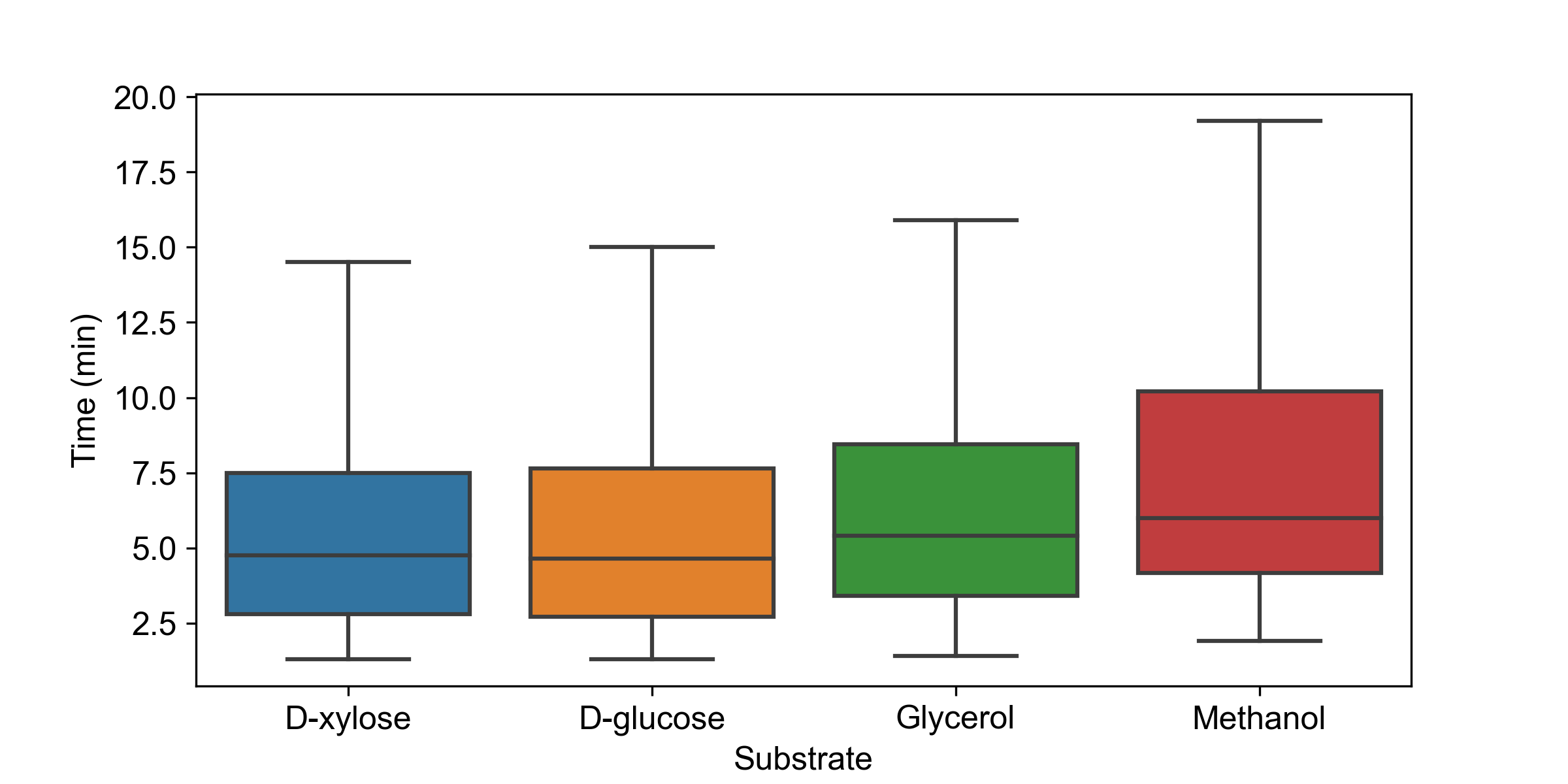


**Figure S7.** Computational time for predicting the synthetic pathways of 300 products from four substrates in *E. coli* using the QHEPath algorithm.

In the QHEPath algorithm, in step 2 of Eq. 6 (${0.1v_{m}^{P}\leq v}_{product}$), the parameter 0.1 indicates that the flux of the target product ($v_{product}$) is not less than 10% of $v_{m}^{P}$. The parameter was set relatively low to introduce fewer heterologous reactions for non-native product synthesis and to obtain more optimized pathways. To evaluate the parameter stability, we varied the parameter in step 2 of Eq. 6 over a range from 0.1 to 1.0. This variation meant that the flux of the target product ($v_{product}$) was not less than 10% to 100% of $v_{m}^{P}$. We then observed the corresponding changes in the minimum number of heterologous reactions (Nsyn) required to achieve the producibility of non-native products. Our results showed that Nsyn remained unchanged between 10% and 30%, with Nsyn being minimized (Figure S8). Therefore, setting the parameter to 10% is reasonable and stable. This test demonstrated the robustness of the QHEPath algorithm.


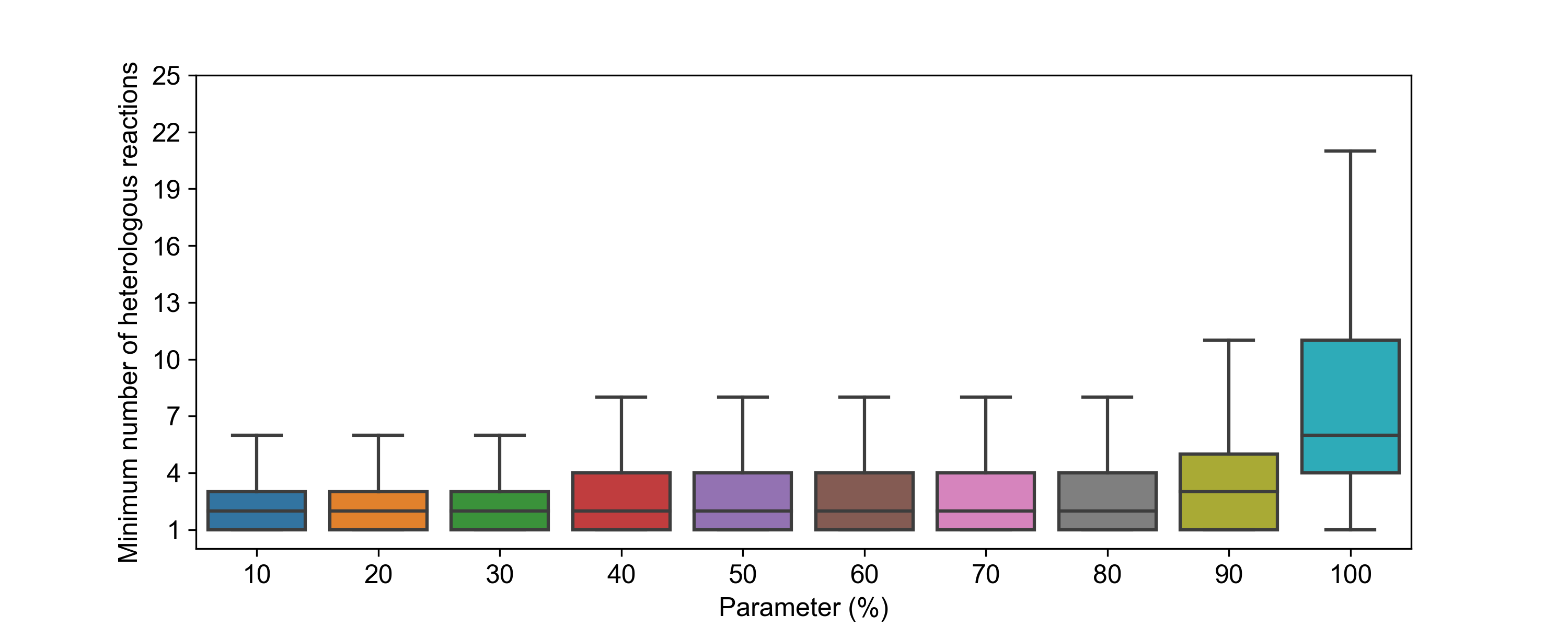


**Figure S8.** In the QHEPath algorithm, the effect of varying the parameter in step 2 of Eq. 6 (${0.1v_{m}^{P}\leq v}_{product}$) over a range from 0.1 to 1.0 on the minimum number of heterologous reactions (Nsyn).

# Note S5

In the synthesis of the non-native product sarcosine in *E. coli*, QHEPath predicted four synthetic pathways. Introducing one heterologous reaction enabled sarcosine production in *E. coli* (Figure S9a). As additional heterologous reactions were introduced, the sarcosine yield increased. With the introduction of two heterologous reactions (P2), the yield of sarcosine reached 95% of $Y_{m}^{P}$, representing a 139% increase compared to pathway P1. Although pathway P4 exhibited a higher yield than P2 and reached $Y_{m}^{P}$, it required the introduction of six heterologous reactions, resulting in only a 5% increase in yield. Similarly, for the synthetic pathways predicted by QHEPath for non-native 4-hydroxy-benzyl alcohol in *E. coli*, introducing two heterologous reactions enabled the synthesis of 4-hydroxy-benzyl alcohol (Figure S9b). As more heterologous reactions were introduced, the yield of 4-hydroxy-benzyl alcohol continued to increase. When 12 heterologous reactions were introduced, the yield reached its $Y_{m}^{P}$. Specifically, with the introduction of five heterologous reactions, the yield of 4-hydroxy-benzyl alcohol reached 97% of $Y_{m}^{P}$, representing a 74% increase compared to pathway P1. These cases demonstrate that the stepwise introduction in the QHEPath algorithm balances yield improvement and the number of heterologous reactions, rather than solely calculating the highest-yield pathway. This balance is crucial for reducing the difficulty of engineering modifications and the cost of enzymes.


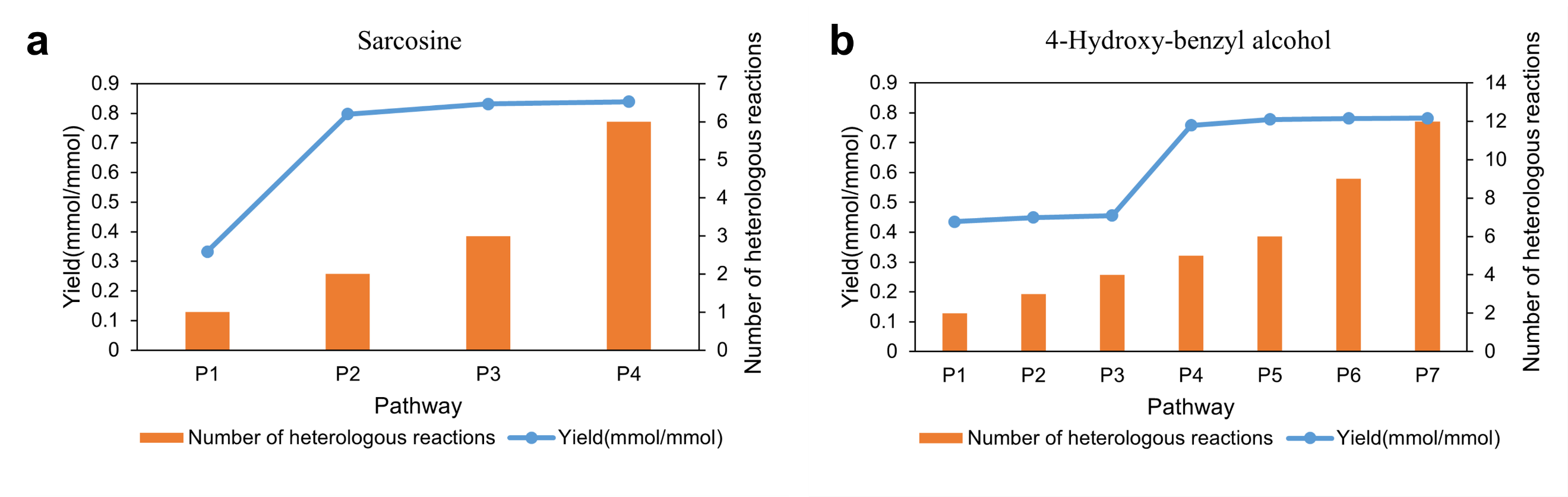


**Figure S9.** QHEPath predicted four synthetic pathways for the non-native product sarcosine (a) and 4-hydroxy-benzyl alcohol (b) with varying yields and numbers of heterologous reactions using *E. coli* as the chassis.

# Note S6

The description below and Supplementary Figure S10-S15 illustrate the pathways predicted by QHEPath, along with comparisons to validated pathways from the literature.

**(1) Acetone**: QHEPath predicts that introducing one heterologous reaction (ADCi: acetoacetate + H^+^ => acetone + CO_2_) enables acetone production in *E. coli* by avoiding the carbon loss during the synthesis of acetyl-CoA from pyruvate (Figure S10a). Building upon this, the incorporation of a non-oxidative glycolysis pathway (NOG) (PKETF: D-fructose 6-phosphate + phosphate --> acetyl phosphate + D-erythrose 4-phosphate + H_2_O) can enhance acetone yield from 1 to 1.5 mol/mol glucose (Figure S10b). The prediction result by QHEPath has been experimentally validated by Yang et al (Figure S10c). They demonstrated that the introduction of the heterologous gene *fxpk*, encoding phosphoketolase, into *E. coli* increased the yield of acetone from 0.38 to 0.47 mol/mol glucose.


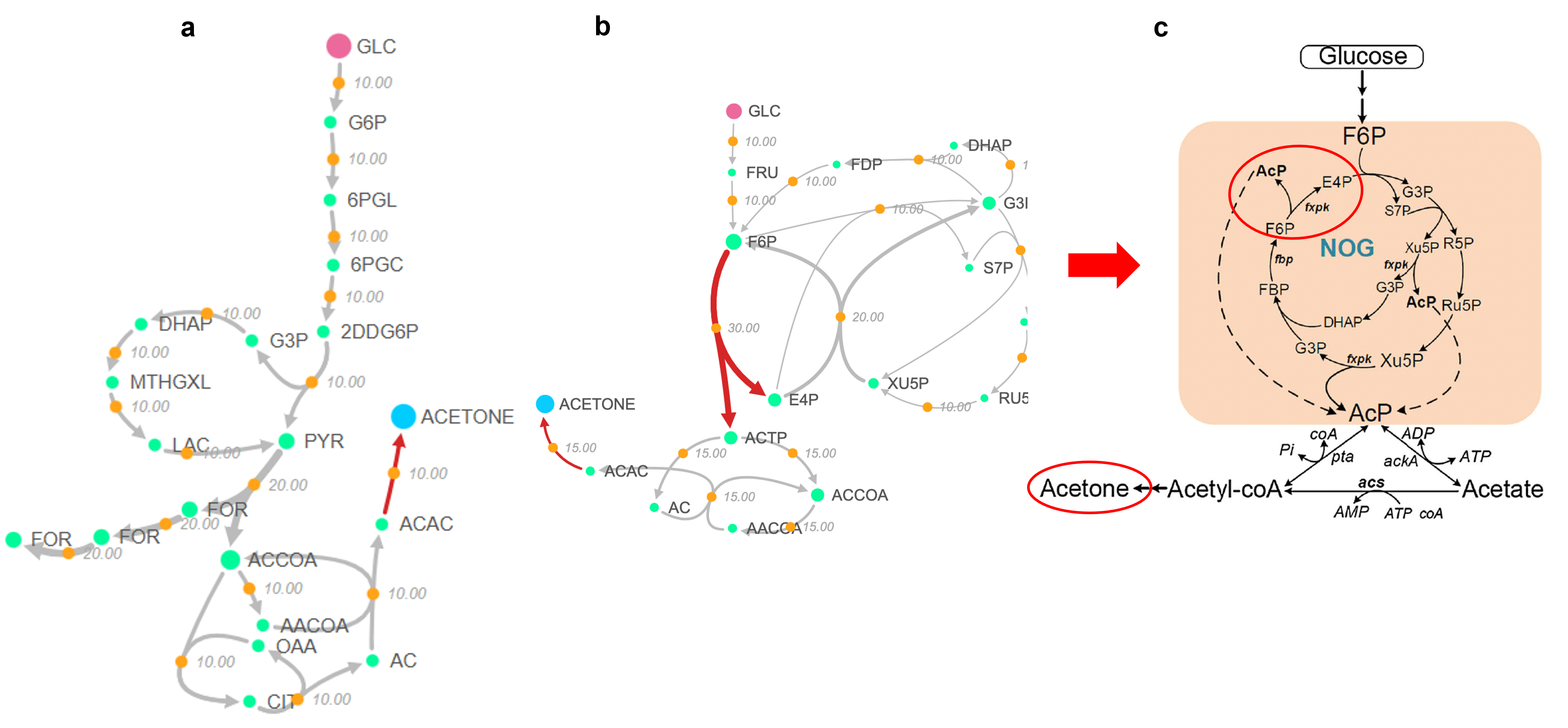


**Figure S10.** The synthetic pathways of acetone predicted by QHEPath and experimentally validated in the literature. (a) The synthesis pathway of acetone predicted by QHEPath in *E. coli* involves the introduction of one heterologous reaction (red lines). (b) The high-yield synthesis pathway of acetone predicted by QHEPath in *E. coli* involves the introduction of two heterologous reactions (red lines). (c) The synthesis pathway of acetone from Figure 1 in the literature ^[4]^. F6P, D-fructose 6-phosphate; E4P, D-erythrose 4-phosphate; ACTP, acetyl phosphate; ACAC, acetoacetate; AcP, acetyl phosphate.

Note: In the NOG pathway, the key enzyme phosphoketolase is a bifunctional enzyme capable of catalyzing reactions PKETF (D-fructose 6-phosphate + phosphate => acetyl phosphate + D-erythrose 4-phosphate + H_2_O) and PKETX (Phosphate + D-Xylulose 5-phosphate => Acetyl phosphate + Glyceraldehyde 3-phosphate + H_2_O). The introduction of either reaction PKETF or PKETX separately, or both reactions simultaneously, can improve the yield of acetone from 1 to 1.5 mol/mol glucose.

**(2)** **3-hydroxypropanoate**: QHEPath predicts that *E. coli* possesses the intact native synthesis pathway of 3-hydroxypropanoate (3HPP), but the pathway is complex, and the yield is low due to decarboxylation of the intermediate carbamate (CBM) and orotidine 5'-phosphate (OROT5P) (Figure S11a). A high-yield synthesis pathway for 3HPP compared to the native pathway is predicted by QHEPath (Figure S11b). This pathway exhibits a 61.7% increase in yield by introducing a heterologous reaction APATr (Beta-Alanine + Pyruvate <=> L-Alanine + Malonate semialdehyde). Not only does the new pathway avoid the decarboxylation of CBM and OROT5P, but it also significantly reduces the number of reaction steps. The prediction result by QHEPath has been experimentally validated by Song et al.^[5]^ (Figure S11c). Based on this experimental evidence, although the 3HPP pathway naturally exists in *E. coli*, the initial strain did not detect 3HPP before the introduction of the heterologous β-alanine pyruvate transaminase, catalyzing reaction APATr. Following the introduction of APATr, the production of 91.9 mg/L 3HPP was achieved.


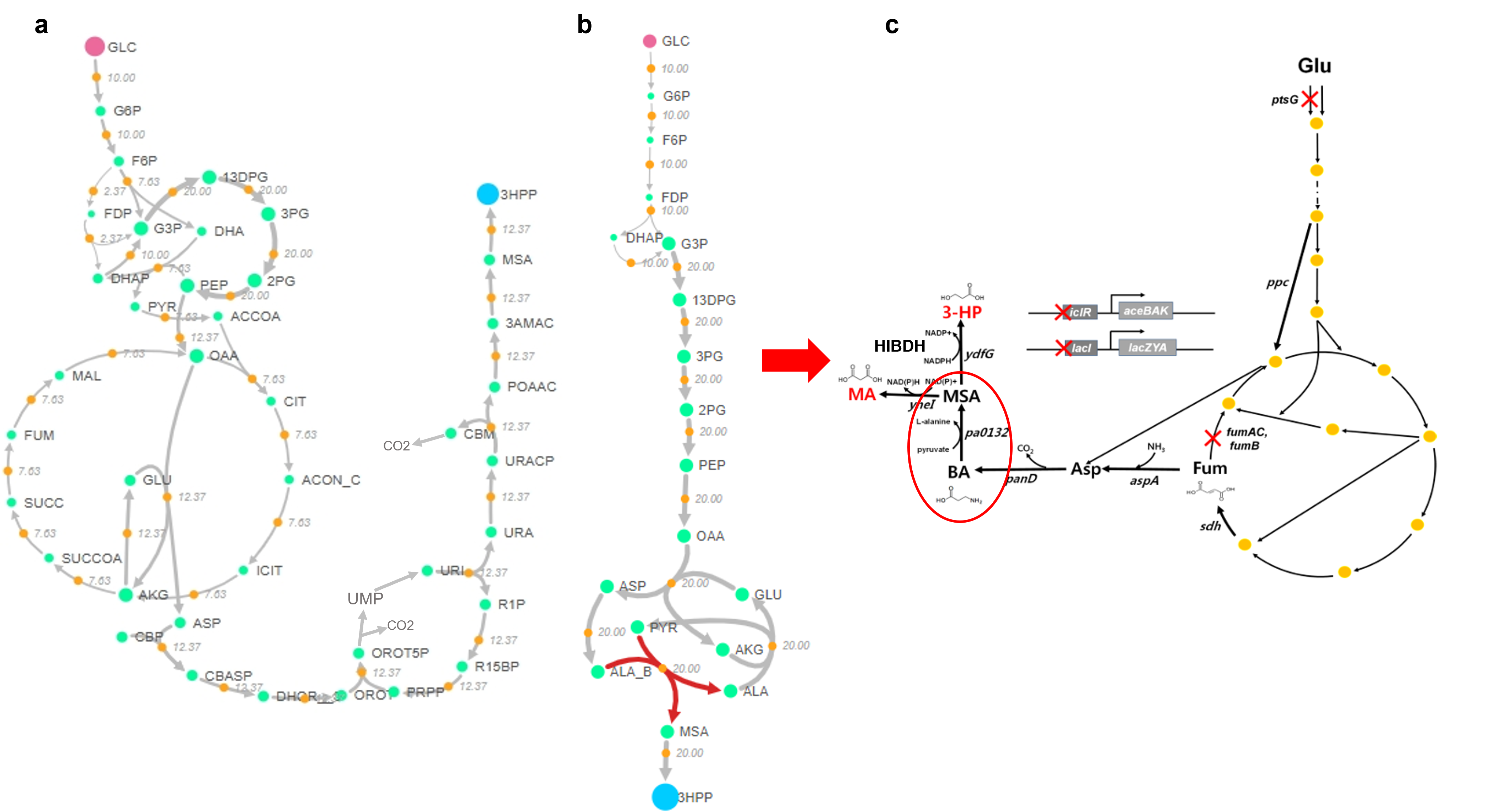
 **Figure S11.** The synthetic pathways of 3-hydroxypropanoate (3HPP) predicted by QHEPath and experimentally validated in the literature. (a) The native synthesis pathway of 3HPP predicted by QHEPath in *E. coli*. (b) The high-yield synthesis pathway of 3HPP predicted by QHEPath in *E. coli* involves introducing one heterologous reaction (red lines). (c) The synthesis pathway of 3HPP from Figure 1 in the literature[5]. PYR, Pyruvate; ALA, L-Alanine; ALA_B, Beta-Alanine; MSA, Malonate semialdehyde, BA, Beta-Alanine.

**(3) Poly(3-hydroxybutyrate) (PHB)**: QHEPath predicts that introducing two heterologous reactions enables the non-native product PHB to be produced in *E. coli* (HBCE: (S)-3-Hydroxybutanoyl-CoA <=> (R)-3-Hydroxybutyryl-CoA; PHBS_syn_1: (R)-3-Hydroxybutyryl-CoA <=> Coenzyme A + PHB) (Figure S12a). Building upon this, the incorporation of a non-oxidative glycolysis pathway (NOG) (PKETX: Phosphate + D-Xylulose 5-phosphate => Acetyl phosphate + Glyceraldehyde 3-phosphate + H_2_O) can enhance PHB yield from 1 to 1.33 mol/mol glucose (Figure S12b). The prediction result by QHEPath has been experimentally validated by Zheng et al. ^[6]^ (Figure S12c). The NOG pathway was introduced into *E. coli* and the yield of PHB in the engineered strain was increased from 0.16 g P3HB/g glucose to 0.24 gPHB/g glucose.


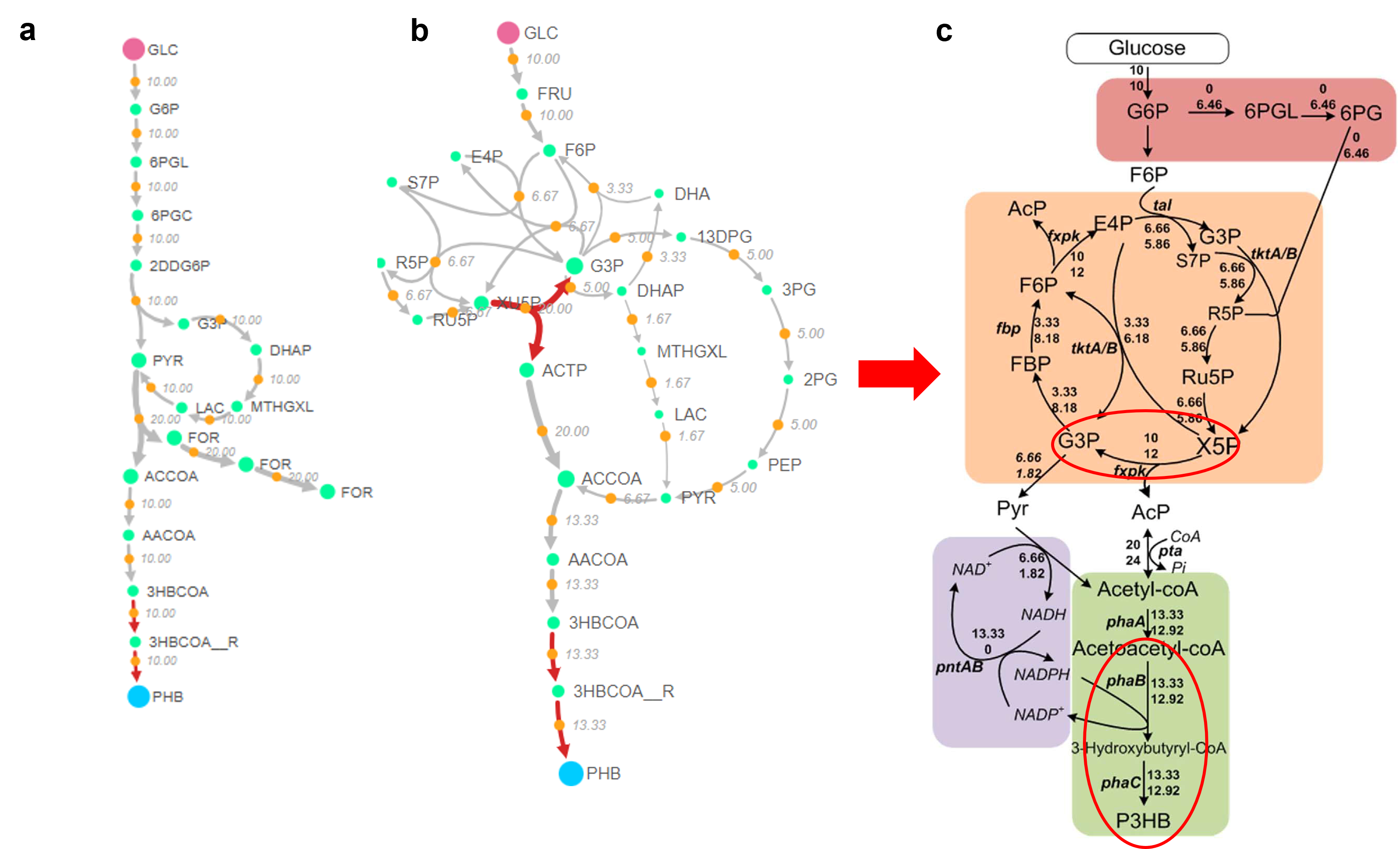
 **Figure S12.** The synthetic pathways of PHB predicted by QHEPath and experimentally validated in the literature. (a) The synthesis pathway of PHB predicted by QHEPath involves the introduction of two heterologous reactions to enable PHB to be synthesized by *E. coli* (red lines). (b) The high-yield synthesis pathway of PHB predicted by QHEPath in *E. coli* involves the introduction of three heterologous reactions (red lines). (c) The synthesis pathway of PHB from Figure 1 in the literature[6]. XU5P, D-Xylulose 5-phosphate; G3P, Glyceraldehyde 3-phosphate; ACTP, acetyl phosphate; 3HBCOA: (S)-3-Hydroxybutanoyl-CoA; 3HBCOA_R: (R)-3-Hydroxybutyryl-CoA.

**(4) L-arginine**: The native pathway for L-arginine synthesis in *E. coli* has a yield of 0.91 mol/mol glucose (Figure S13a). QHEPath predicts that introducing one heterologous reaction catalyzed by ornithine acetyltransferase (ORNTAC: N-Acetylornithine + L-Glutamate <=> N-Acetyl-L-glutamate + L-Ornithine) can improve the yield to 0.95 mol/mol glucose (Figure S13b)*.* The prediction result by QHEPath has been experimentally validated by Wang et al*.*^[7]^ (Figure S13c). The heterologous gene *argJ* encoding ornithine acetyltransferase was introduced into *E. coli,* demonstrating that the heterologous reaction ORNTAC effectively increased the yield of L-arginine.


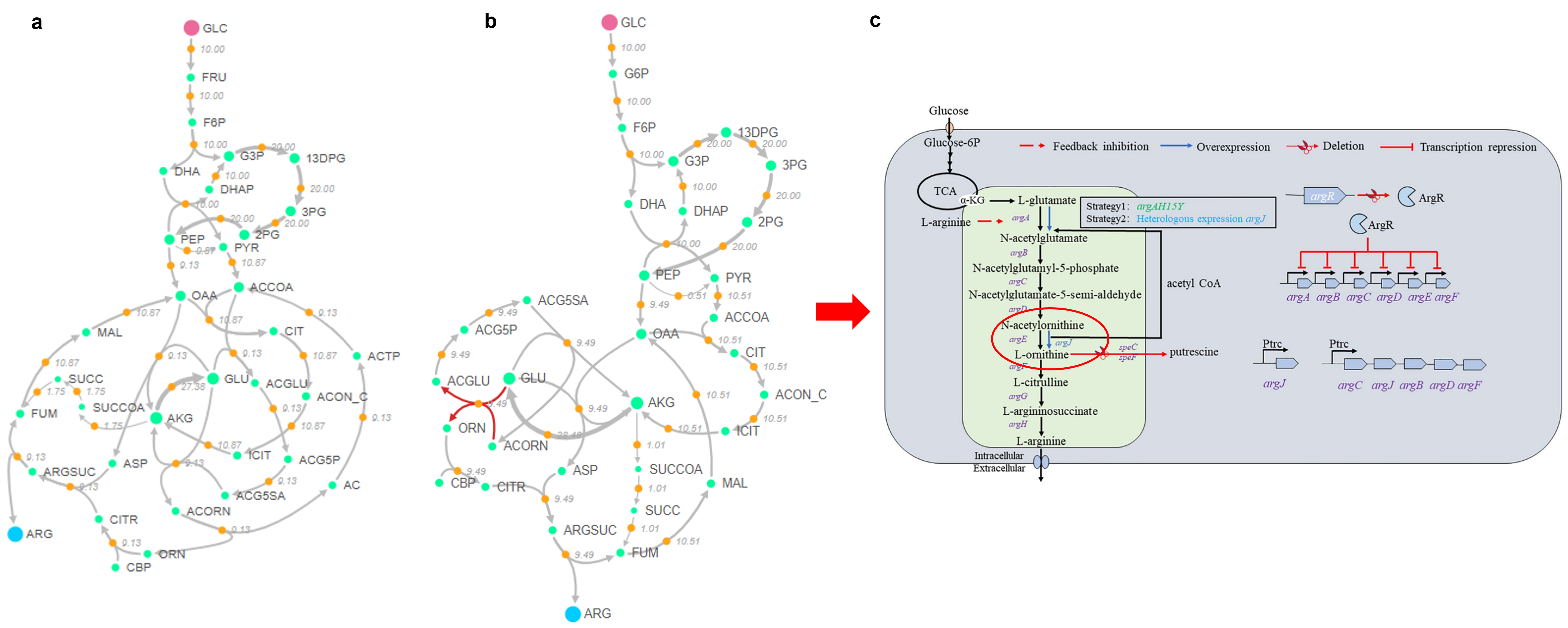


**Figure S13.** The synthetic pathways of L-arginine predicted by QHEPath and experimentally validated in the literature. (a) The native synthesis pathway of L-arginine predicted by QHEPath in *E. coli*. (b) The high-yield pathway of L-arginine predicted by QHEPath involves the introduction of one heterologous reaction (red lines). (c) The synthesis pathway of PHB from Figure 2 in the literature ^[7]^. ACORN: N-Acetylornithine; GLU: L-Glutamate; ACGLU: N-Acetyl-L-glutamate; ORN: L-Ornithine.

**(5) Spermidine**: Spermidine can be synthesized endogenously in yeast. QHEPath predicted that the spermidine yield can be improved in yeast by introducing two heterologous reactions catalyzed by carboxyspermidine dehydrogenase (CASDH) and carboxynorspermidine decarboxylase (CASDC) (CSPMDDH: L-Aspartate 4-semialdehyde + H^+^ + NADPH + Putrescine => Carboxyspermidine + H_2_O + NADP; CSPMDDC: Carboxyspermidine + H^+^ => CO_2_+ Spermidine) (Figure S14a). This prediction result is supported by the experimental results reported by Qin *et al*.[8] (Figure S14b). They introduced two heterologous enzymes CASDH and CASDC from *Vibrio cholera* into yeast, which led to the production of 69 mg/L spermidine, representing a 41% increase compared to the parental strain. Additionally, this study integrated other metabolic engineering strategies, such as gene deletion, overexpression, and attenuation, to further enhance experimental yields.


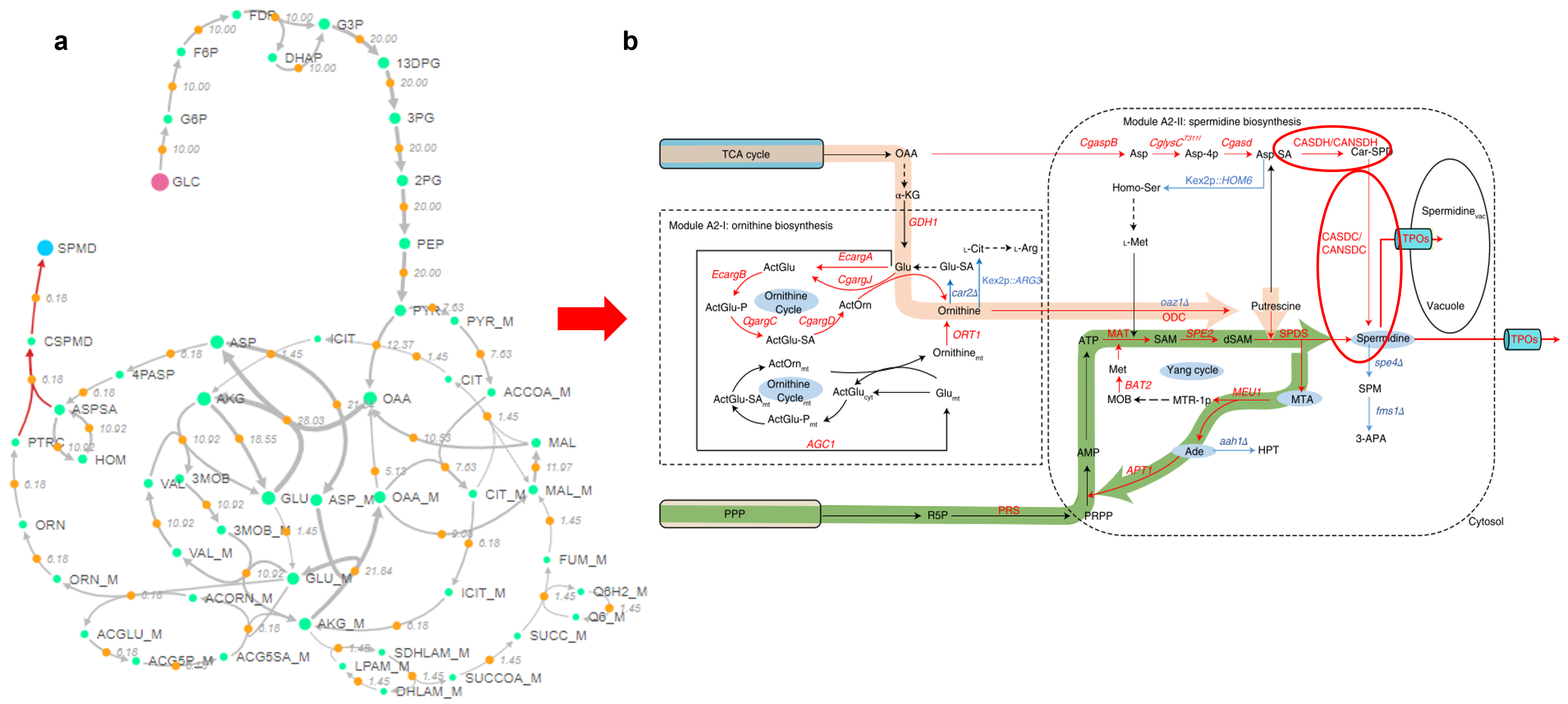


**Figure S14.** The synthetic pathways of spermidine predicted by QHEPath and experimentally validated in the literature. (a) The synthetic pathway of spermidine predicted by QHEPath in yeast involves the introduction of two heterologous reactions (red lines). (b) The synthesis pathway of spermidine from Figure 2 in the literature[8]. ASPSA, L-Aspartate 4-semialdehyde; PTRC, Putrescine; CSPMD, Carboxyspermidine; SPMD, Spermidine; Asp-SA, L-Aspartate 4-semialdehyde, Car-SPD, Carboxyspermidine.

**(6) Farnesene**: QHEPath predicted an increase in the yield of farnesene through the introduction of two heterologous reactions (PKETX: Phosphate + D-Xylulose 5-phosphate => Acetyl phosphate + Glyceraldehyde 3-phosphate + H_2_O; PTAr, Acetyl-CoA + Phosphate <=> Acetyl phosphate + CoA) (Figure S15b). The prediction result by QHEPath has been experimentally validated by Meadows et al.^[9]^ (Supplementary Figure S15c). They introduced two heterologous reactions into yeast and increased farnesene productivity to 5.2 g/L/h from 2.71 g/L/h. The experiments conducted by Meadows et al. demonstrated that QHEPath can predict effective strategies for heterologous introduction, but further genetic engineering modifications are needed to achieve a higher yield and titer.


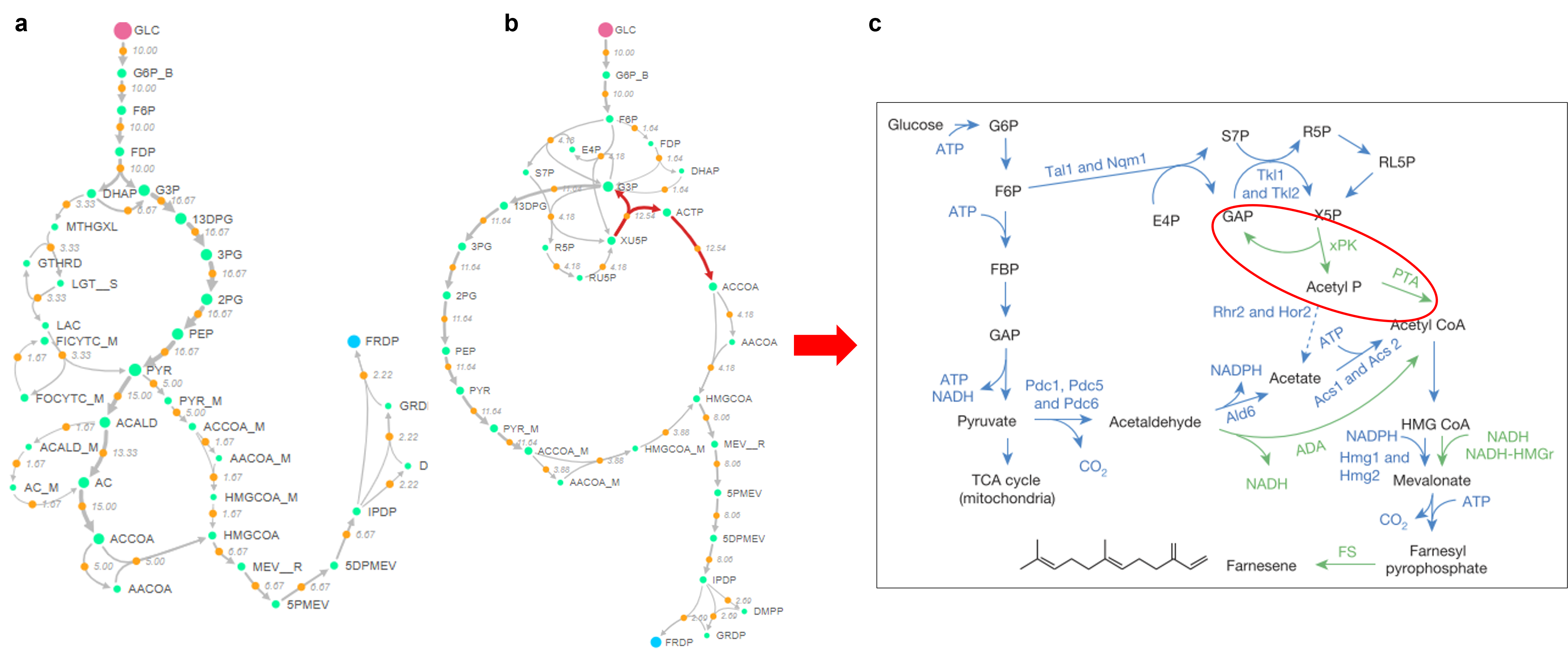
 **Figure S15.** The synthetic pathways of farnesene predicted by QHEPath and experimentally validated in the literature. (a) The native synthesis pathway of farnesene predicted by QHEPath in yeast. (b) The high-yield pathway of farnesene predicted by QHEPath in yeast involves the introduction of two heterologous reactions. (c) The synthesis pathway of spermidine from Figure 1 in the literature [9]. Note: Given that the BiGG database lacks the metabolite farnesene, and farnesene is directly derived from farnesyl diphosphate through a single-step dephosphorylation reaction catalyzed by farnesene synthase, farnesyl diphosphate is used as the target product in the simulation. XU5P, D-Xylulose 5-phosphate; G3P, Glyceraldehyde 3-phosphate; ACTP, Acetyl phosphate; X5P, D-Xylulose 5-phosphate; GAP, Glyceraldehyde 3-phosphate.

# Note S7

QHEPath is a cloud-based, serverless web tool for quantitative heterologous pathway design. It relies on a quality-controlled universal model from the BiGG database and employs an algorithm to compute multiple suboptimal and optimal pathways for product synthesis. It can be used to calculate and visualize multiple product optimization pathways with higher yields for different substrates, products, and multiple host organisms including 108 GEMs from 35 different species. Additionally, the platform supports the integration of 13 summarized pathway optimization strategies, spanning carbon-conserving and energy-conserving approaches, into the host organism. This integration allows for the evaluation of these strategies to potentially enhance the yield of the target product.

1. Pathway Calculation
2. Parameter setting

Users can search and select the genome-scale metabolic network model (GEM) of host organism through entering the model ID or strain name in the ‘Model’ box. The substrate and target product can be set by inputting metabolite name or ID in BiGG, KEGG or MetaCyc in the ‘Substrate’ and ‘Product’ box. The parameter ‘Number of optimization’ represents the option to conduct multiple rounds of optimization to obtain additional optimized pathways. Users can adjust this parameter to decrease or increase the calculation time or obtain more pathways. It is essential to note that the value must be an integer. Users provide email to remind them when the calculations are complete. After all the parameters are set, the task can be submitted by clicking the ‘Submit’ button (Figure S16).


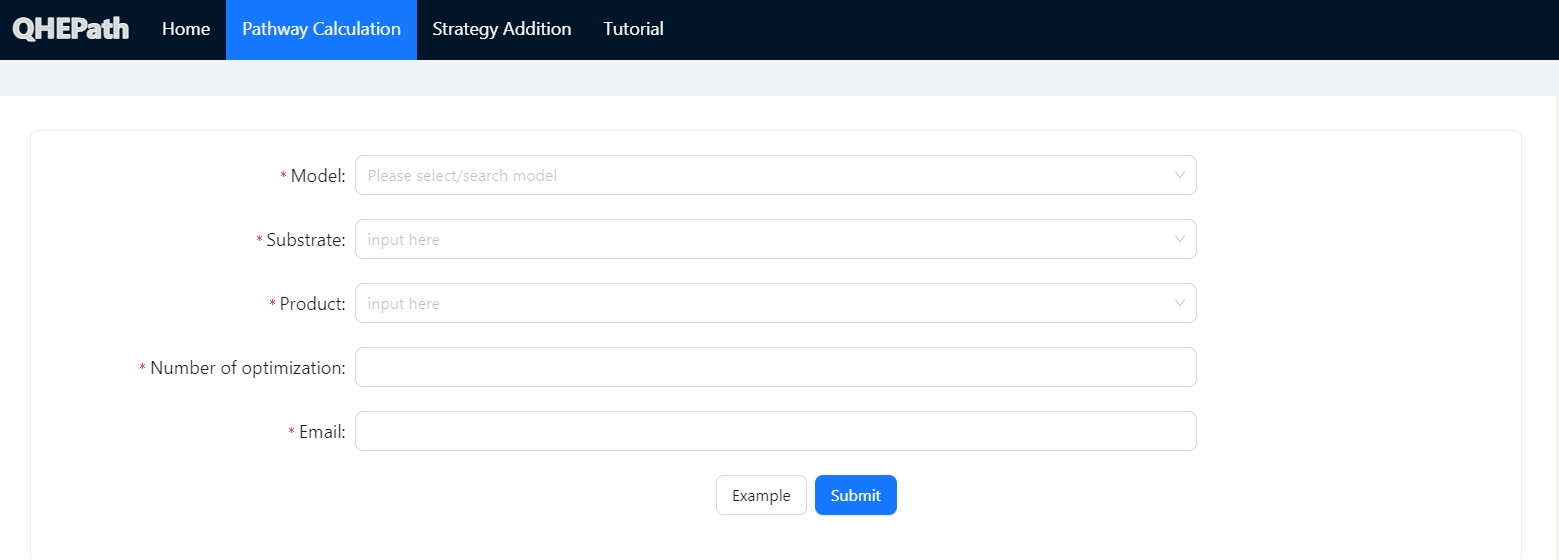


**Figure S16.** The parameters setting for pathway calculations.

1. Visualization and download of results

After the task that meets the requirements is submitted, the program will jump to the ‘Results’ page. The page shows all the settings and the task status. Once the task is complete, the status is ‘Finished’, and an email will be sent to the provided email address.


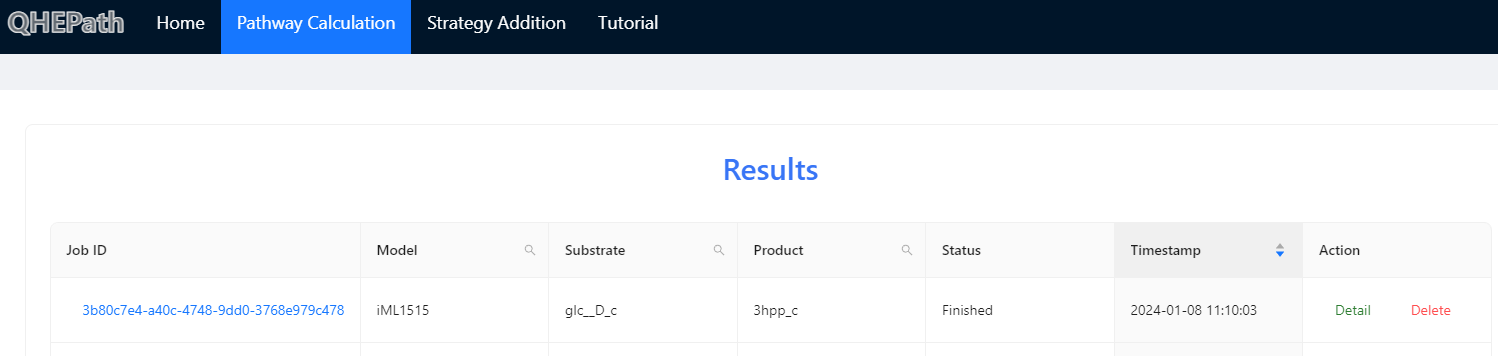


**Figure S17.** The results page.

The details of each design task can be displayed by clicking the ‘Job ID’ link (Figure S17). The 'Details' page displays the synthetic pathway of the target product, the number of heterologous reactions, and the product yield (Figure S17). There is a default sort, and users can reorder as required. Each pathway can be visualized by clicking the ‘Visualization’ button and can be downloaded by clicking the ‘Download’ button (Figure S18).


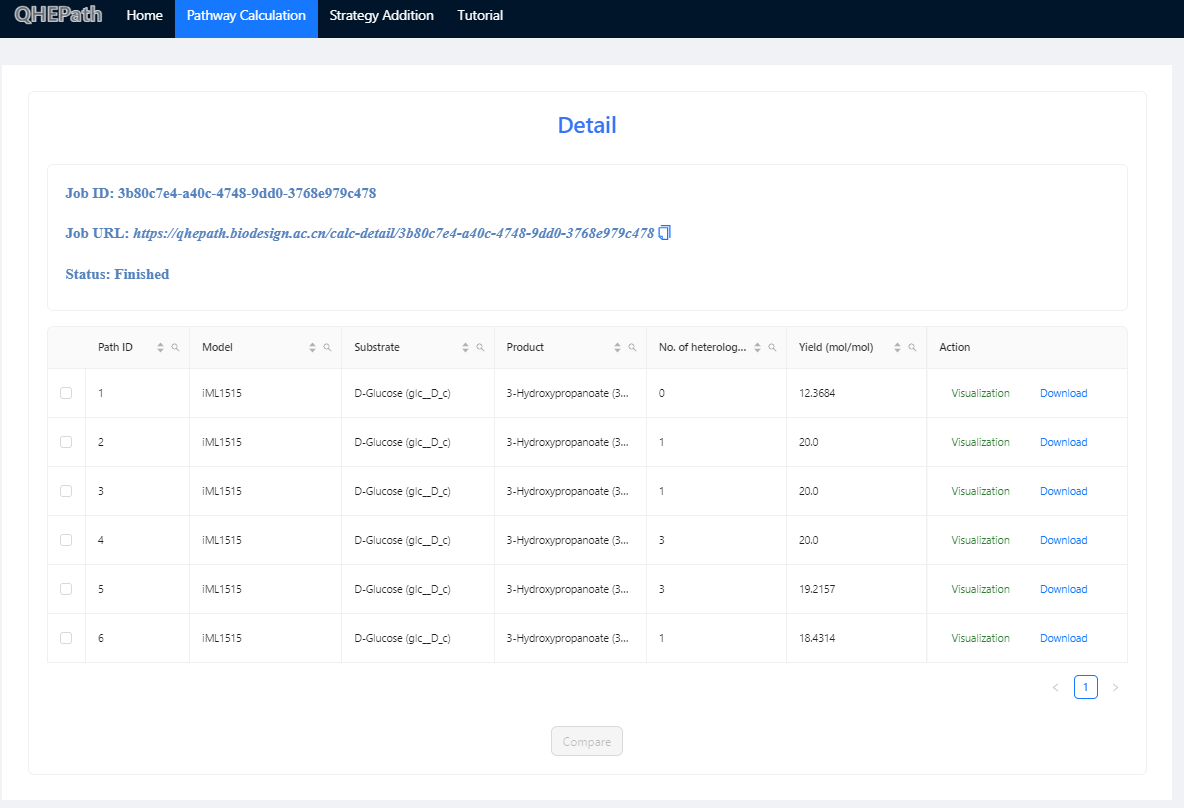
 **Figure S18.** The details page.

After clicking the ‘Visualization’ button, users can customize whether to display cofactors and "Submit" the pathway for visualization (Figure S19). Users can select "Show Reaction Nodes" and adjust the visualization results by dragging the nodes and edges in the diagram. The generated pathway map can be saved in JSON or SVG format, or the coordinates of each node can be exported directly to facilitate pathway map reproduction. When double-clicking on nodes and edges in the diagram, detailed annotated information about the metabolites and reactions is provided, such as reaction names, equations, fluxes, and EC number. In the synthetic pathways of products, grey lines represent native reactions in the host, while red lines represent heterologous reactions. Additionally, users can conduct a comparative analysis of the calculated results and visualize the differences by selecting two or three pathways.


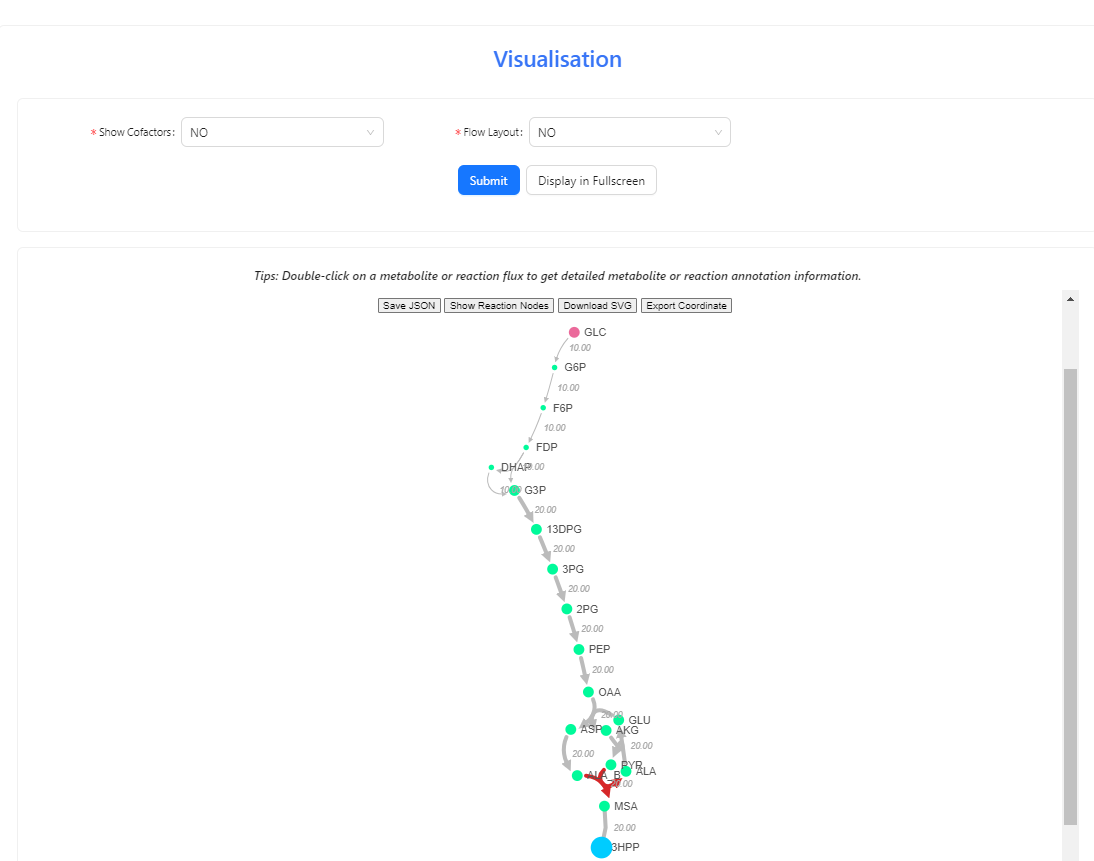


**Figure S19.** The visualization page.

1. Strategy Addition
2. Parameter setting

In the ‘Strategy Addition’ module, users can select the summarized 13 pathway optimization strategies including carbon-conserving and energy-conserving into the host organism to evaluate these strategies for the potential enhancement of the target product's yield. Users can search and select the genome-scale metabolic network model (GEM) of host organism through entering the model ID or strain name in the ‘Model’ box. The substrate and target product can be set by inputting metabolite name or ID in BiGG, KEGG or MetaCyc in the ‘Substrate’ and ‘Product’ box. The 13 summarized pathway optimization strategies need to be selected to for addition to the host model. Users provide email to remind them when the calculations are complete. After all the parameters are set, the task can be submitted by clicking the ‘Submit’ button (Figure S20).


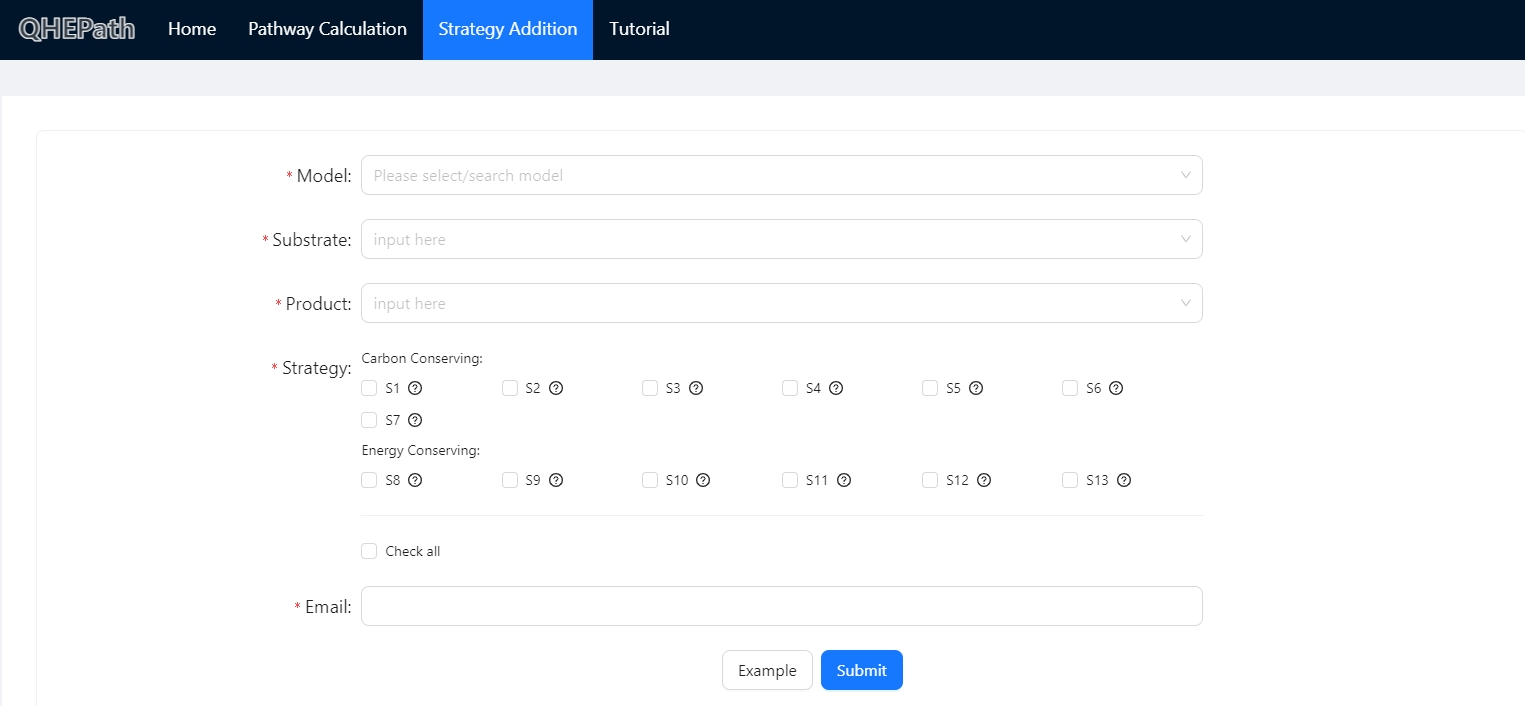


**Figure S20.** The parameters setting for strategy addition.

1. Visualization and download of results

After the task that meets the requirements is submitted, the program will jump to the ‘Results’ page. The page shows all the settings and the task status. Once the task is complete, the status is ‘Finished’, and an email will be sent to the provided email address.


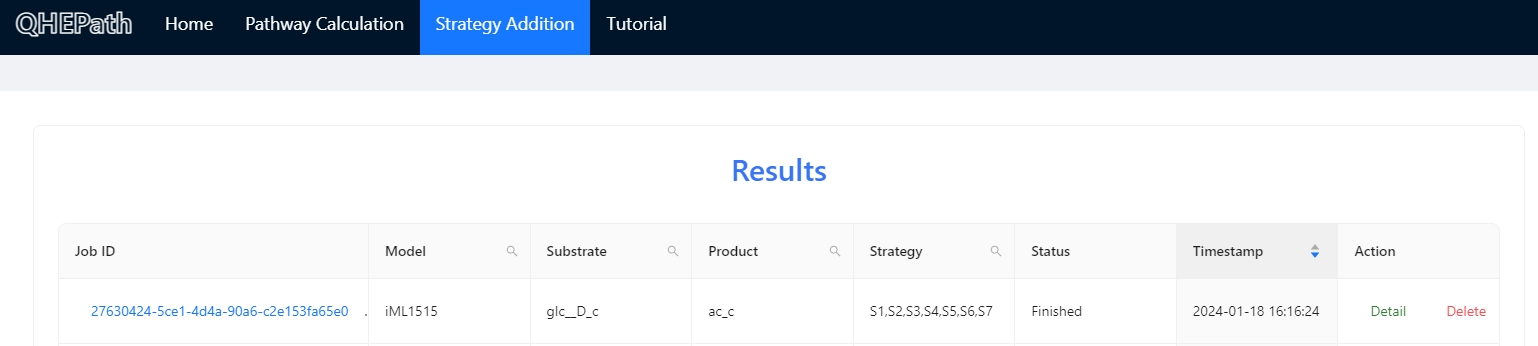


**Figure S21.** The results page.

The details of each design task can be displayed by clicking the ‘Job ID’ link (Figure S21). The ‘Details’ page displays the yield of the target product after integrating these selected strategies (Figure S22). There is a default sort, and users can reorder as required. Each pathway can be visualized by clicking the ‘Visualization’ button and can be downloaded by clicking the ‘Download’ button (Figure S22).


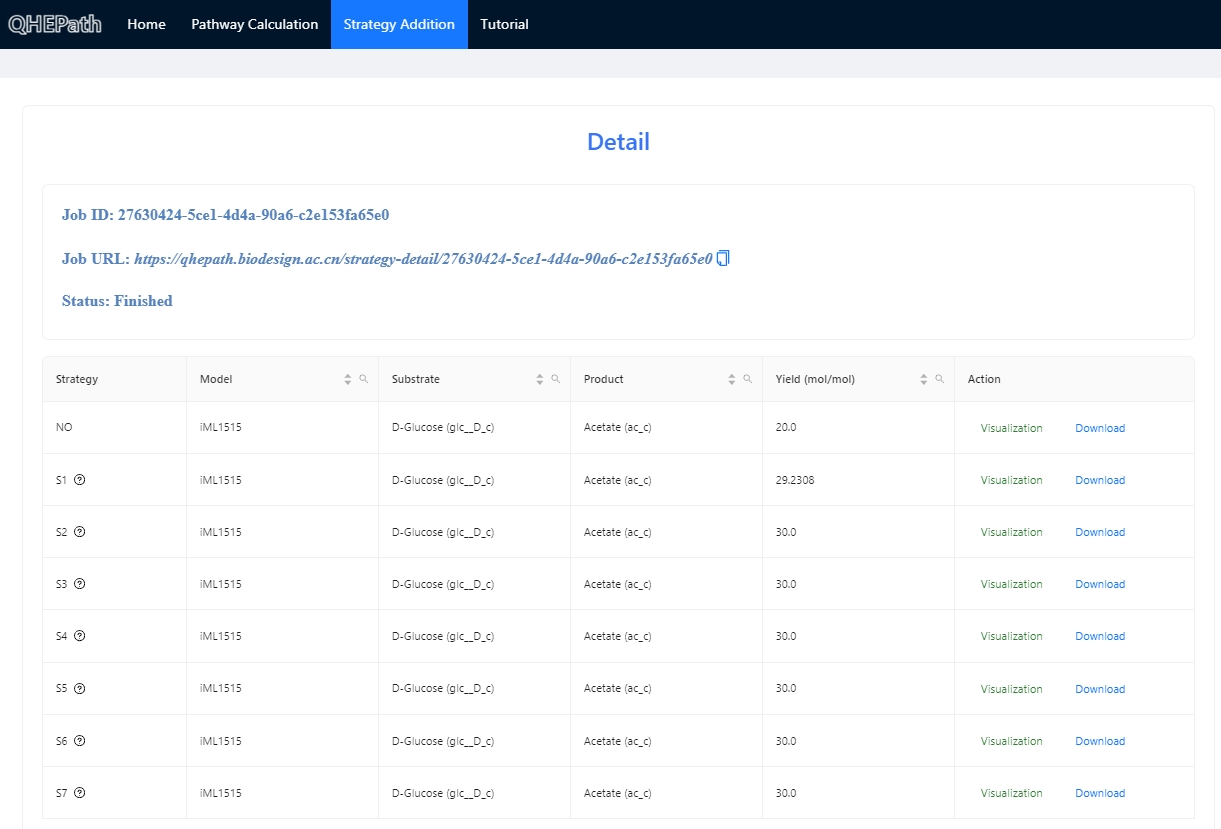


**Figure S22.** The details page.

After clicking the ‘Visualization’ button, users can customize whether to display cofactors and "Submit" the pathway for visualization (Figure S23). Users can select "Show Reaction Nodes" and adjust the visualization results by dragging the nodes and edges in the diagram. The generated pathway map can be saved in JSON or SVG format, or the coordinates of each node can be exported directly to facilitate pathway map reproduction. When double-clicking on nodes and edges in the diagram, detailed annotated information about the metabolites and reactions is provided, such as reaction names, equations, fluxes, and EC number. In the synthetic pathways of products, grey lines represent native reactions in the host, while red lines represent heterologous reactions. Additionally, users can conduct a comparative analysis of the calculated results and visualize the differences by selecting two or three pathways.


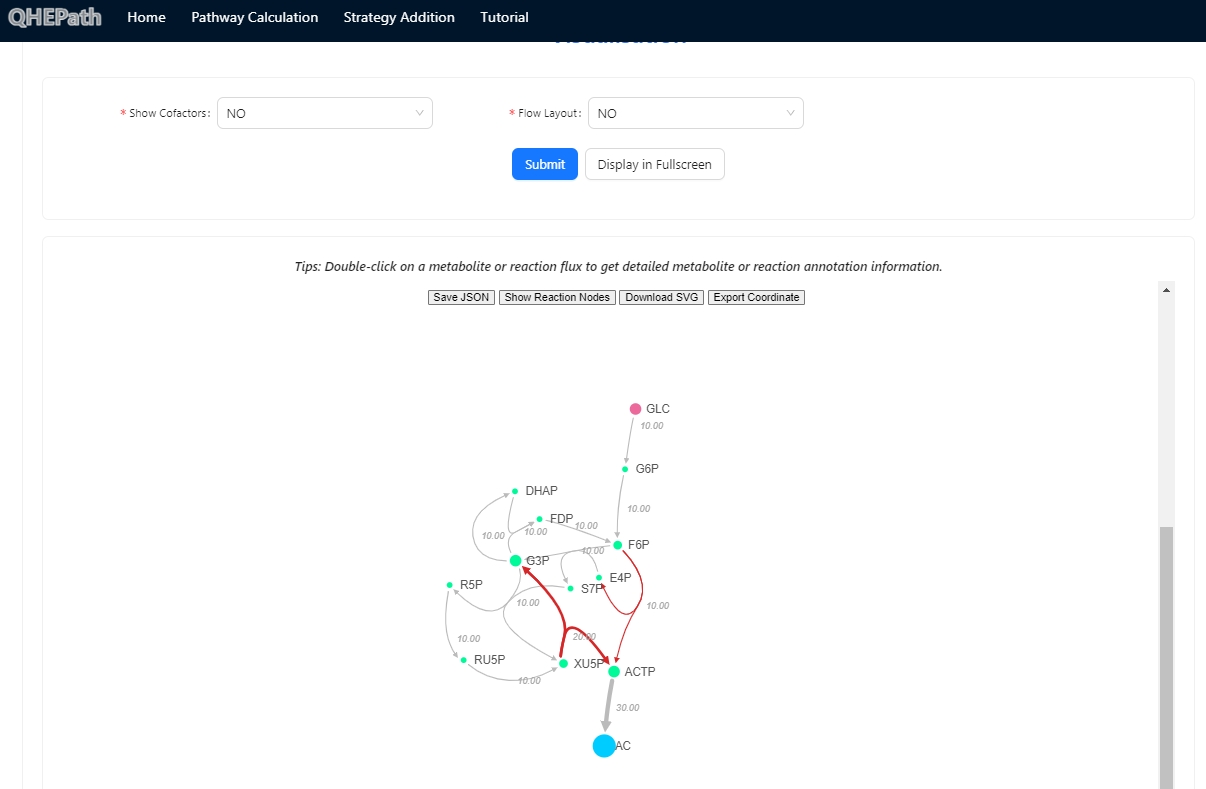


**Figure S23.** The visualization page.

# References

[1] C. Lieven, M. E. Beber, B. G. Olivier, F. T. Bergmann, M. Ataman, P. Babaei, J. A. Bartell, L. M. Blank, S. Chauhan, K. Correia, C. Diener, A. Drager, B. E. Ebert, J. N. Edirisinghe, J. P. Faria, A. M. Feist, G. Fengos, R. M. T. Fleming, B. Garcia-Jimenez, V. Hatzimanikatis, W. van Helvoirt, C. S. Henry, H. Hermjakob, M. J. Herrgard, A. Kaafarani, H. U. Kim, Z. King, S. Klamt, E. Klipp, J. J. Koehorst, M. Konig, M. Lakshmanan, D. Y. Lee, S. Y. Lee, S. Lee, N. E. Lewis, F. Liu, H. Ma, D. Machado, R. Mahadevan, P. Maia, A. Mardinoglu, G. L. Medlock, J. M. Monk, J. Nielsen, L. K. Nielsen, J. Nogales, I. Nookaew, B. O. Palsson, J. A. Papin, K. R. Patil, M. Poolman, N. D. Price, O. Resendis-Antonio, A. Richelle, I. Rocha, B. J. Sanchez, P. J. Schaap, R. S. Malik Sheriff, S. Shoaie, N. Sonnenschein, B. Teusink, P. Vilaca, J. O. Vik, J. A. H. Wodke, J. C. Xavier, Q. Yuan, M. Zakhartsev, C. Zhang, *Nat Biotechnol* **2020**, *38* (3), 272, <https://doi.org/10.1038/s41587-020-0446-y>.

[2] C. J. Fritzemeier, D. Hartleb, B. Szappanos, B. Papp, M. J. Lercher, *PLoS Comput Biol* **2017**, *13* (4), e1005494, <https://doi.org/10.1371/journal.pcbi.1005494>.

[3] T. Kanno, K. Kasai, Y. Ikejiri-Kanno, K. Wakasa, Y. Tozawa, *Plant Mol Biol* **2004**, *54* (1), 11, <https://doi.org/10.1023/B:PLAN.0000028729.79034.07>.

[4] X. Yang, Q. Yuan, Y. Zheng, H. Ma, T. Chen, X. Zhao, *Biotechnol Lett* **2016**, *38* (8), 1359, <https://doi.org/10.1007/s10529-016-2115-2>.

[5] C. W. Song, J. W. Kim, I. J. Cho, S. Y. Lee, *ACS Synth Biol* **2016**, *5* (11), 1256, <https://doi.org/10.1021/acssynbio.6b00007>.

[6] Y. Zheng, Q. Yuan, X. Yang, H. Ma, *Enzyme and Microbial Technology* **2017**, *106*, 60, <https://doi.org/10.1016/j.enzmictec.2017.07.003>.

[7] H.-D. Wang, J.-Z. Xu, W.-G. Zhang, *Applied Microbiology and Biotechnology* **2022**, *106* (17), 5603, <https://doi.org/10.1007/s00253-022-12109-4>.

[8] J. Qin, A. Krivoruchko, B. Ji, Y. Chen, M. Kristensen, E. Özdemir, J. D. Keasling, M. K. Jensen, J. Nielsen, *Nature Catalysis* **2021**, *4* (6), 498, <https://doi.org/10.1038/s41929-021-00631-z>.

[9] A. L. Meadows, K. M. Hawkins, Y. Tsegaye, E. Antipov, Y. Kim, L. Raetz, R. H. Dahl, A. Tai, T. Mahatdejkul-Meadows, L. Xu, L. Zhao, M. S. Dasika, A. Murarka, J. Lenihan, D. Eng, J. S. Leng, C. L. Liu, J. W. Wenger, H. Jiang, L. Chao, P. Westfall, J. Lai, S. Ganesan, P. Jackson, R. Mans, D. Platt, C. D. Reeves, P. R. Saija, G. Wichmann, V. F. Holmes, K. Benjamin, P. W. Hill, T. S. Gardner, A. E. Tsong, *Nature* **2016**, *537* (7622), 694, <https://doi.org/10.1038/nature19769>.
